# Elucidation of the BMI1 interactome identifies novel regulatory roles in glioblastoma

**DOI:** 10.1093/narcan/zcab009

**Published:** 2021-03-22

**Authors:** Verónica Freire-Benéitez, Nicola Pomella, Thomas O Millner, Anaëlle A Dumas, Maria Victoria Niklison-Chirou, Eleni Maniati, Jun Wang, Vinothini Rajeeve, Pedro Cutillas, Silvia Marino

**Affiliations:** Blizard Institute, Barts and the London School of Medicine and Dentistry, Queen Mary University of London, E1 2AT, London, UK; Blizard Institute, Barts and the London School of Medicine and Dentistry, Queen Mary University of London, E1 2AT, London, UK; Blizard Institute, Barts and the London School of Medicine and Dentistry, Queen Mary University of London, E1 2AT, London, UK; Blizard Institute, Barts and the London School of Medicine and Dentistry, Queen Mary University of London, E1 2AT, London, UK; Blizard Institute, Barts and the London School of Medicine and Dentistry, Queen Mary University of London, E1 2AT, London, UK; Centre for Therapeutic Innovation (CTI-Bath), Department of Pharmacy and Pharmacology, University of Bath, Bath BA2 7AY, UK; Barts Cancer Institute, Barts and the London School of Medicine and Dentistry, Queen Mary University of London, London EC1M 6AS UK; Barts Cancer Institute, Barts and the London School of Medicine and Dentistry, Queen Mary University of London, London EC1M 6AS UK; Barts Cancer Institute, Barts and the London School of Medicine and Dentistry, Queen Mary University of London, London EC1M 6AS UK; Barts Cancer Institute, Barts and the London School of Medicine and Dentistry, Queen Mary University of London, London EC1M 6AS UK; Blizard Institute, Barts and the London School of Medicine and Dentistry, Queen Mary University of London, E1 2AT, London, UK

## Abstract

Glioblastoma (GBM) is the most common and aggressive intrinsic brain tumour in adults. Epigenetic mechanisms controlling normal brain development are often dysregulated in GBM. Among these, BMI1, a structural component of the Polycomb Repressive Complex 1 (PRC1), which promotes the H2AK119ub catalytic activity of Ring1B, is upregulated in GBM and its tumorigenic role has been shown *in vitro* and *in vivo*. Here, we have used protein and chromatin immunoprecipitation followed by mass spectrometry (MS) analysis to elucidate the protein composition of PRC1 in GBM and transcriptional silencing of defining interactors in primary patient-derived GIC lines to assess their functional impact on GBM biology. We identify novel regulatory functions in mRNA splicing and cholesterol transport which could represent novel targetable mechanisms in GBM.

## INTRODUCTION

Glioblastoma (GBM) is the most common aggressive intrinsic brain tumour in adults. It extensively infiltrates the surrounding brain tissue, making complete surgical resection impossible. Moreover, GBM displays remarkable resistance to radio- and chemotherapy, which leads to tumour recurrence (1,2). Defining mutations in the isocitrate dehydrogenase genes have been reported in a subset of GBM; however, the majority of cases display an IDH wild-type genotype (3). Molecular subtypes of GBM have been identified (4,5) and single cell transcriptomic data have recently revealed that multiple subtypes can exist within a single tumour, underscoring a high level of inter- and intra-tumour heterogeneity, which significantly contributes to therapeutic resistance (6). Consequently, its prognosis is very poor, with a median survival of only 14 months (7).

Epigenetic mechanisms play a key role in the pathogenesis of GBM (8) but the molecular machinery that underpin this is still poorly understood. Polycomb Group (PcG) proteins are essential epigenetic factors regulating chromatin accessibility and gene expression in various stem cell populations during embryonic development and tissue homeostasis (9) and are often deregulated in cancer (10,11). PcG proteins repress gene expression via two multi-subunit complexes termed Polycomb Repressive complexes (PRC1 and PRC2). PRC2 promotes trimethylation of histone H3 at lysine 27 (H3K27me3), which acts as the epigenetic mark recognized by PRC1. PRC1 then catalyses the monoubiquitylation of histone H2A at lysine 119 (H2AK119ub) impairing transcription elongation (12), as well as promoting chromatin compaction and reducing nucleosomal turnover (13), leading to gene silencing (14). However, gene expression can also be enabled by PcG proteins, for example Ring1B facilitates the topological interaction of the Meis2 promoter with a midbrain-specific enhancer (MBE) within the gene, thus enabling the transition from a repressive to an active state of Meis2 expression during midbrain development (15).

The highly variable modular protein composition of the PRC explains their complex and diverse functional roles. There are canonical (cPRC1) and non-canonical (ncPRC1) sub-complexes of PRC1, where the core RING-PCGF heterodimer is conserved, and the other proteins are variable (16). The core RING-PCGF heterodimer consists of RING1 or RING2, to which one of the six alternative PCGF1–6 is bound. In cPRC1, PHC and CBX subunits bind to this core heterodimer (17–19), while in ncPRC1 Ring1 and YY1 binding protein (RYBP) replace PHC and CBX (20,21). Moreover, the composition of PRC1 sub-complexes also varies depending on the cell type and the cellular differentiation state (10,19) and this diversity plays an important role in mediating PRC1 functional outcomes. For example, a CBX7 to CBX8 switch allows transcriptional activation of differentiation genes, despite persisting H3K27me3 and H2AK119ub marks, in mouse embryonic stem cells (22).

PRC1 components are highly dysregulated in cancer ^8^. cPRC1 members have been shown to be upregulated in breast cancer cells regulating the expression of oncogenic active enhancers such as oestrogen receptor alpha (ERα) and BRD4-containing enhancers in triple-negative breast cancer (TNBC) (23). Moreover, deregulation of ncPRC1 (ncPRC1) proteins also plays a key role in cancer. Downregulation of the lysine demethylase KDM2, member of the ncPRC1.1, reduced cell proliferation *in vitro* and leukemogenesis in humanized xenograft models. This ncPRC1.1 showed binding to loci lacking H3K27me3, which indicates a role of ncPRC1.1 independent of PRC2 (21). However, PcG proteins can also regulate the expression and function of oncogenes and tumour suppressor genes in a PRC-independent manner (24). In prostate cancer, for example, BMI1 (PCGF4) binds to the androgen receptor (AR) independently of the PRC1 complex thereby preventing protein degradation, which results in sustained AR signalling promoting tumour growth (25).

BMI1 overexpression has been reported in many cancers (26), where it controls proliferation and migration of neoplastic cells and also promotes apoptosis; its pharmacological inhibition increases sensitivity to chemotherapy treatments in some cancer types (26–31). BMI1 regulates development and homeostasis of the mammalian central nervous system (CNS) via maintenance of embryonic and adult neural stem cell (NSC) self-renewal (32-34), and it is highly expressed in glioblastoma initiating cells (GIC) (35), the key cellular driver of tumour initiation and maintenance in GBM (36). However, in GBM it is becoming increasingly clear that the functional role of the PRC complexes is highly dependent on both protein composition and cellular context; for example, BMI1 promotes survival of GIC of the mesenchymal subtype while EZH2 plays a similar role in GIC of the proneural subtype (37). At the mechanistic level, BMI1 interacts with RING1A/B leading to stabilization of the complex and increased H2A ubiquitination activity (35,38,39), although mediation of protein–protein interactions leading to a favourable nucleosomal configuration rather than enhancing enzymatic activity is what is believed to predominantly lead to transcriptional repression and oncogenic activity (40–42). PRC1 protein composition has never been characterized in GBM, nor has the impact of fluctuation of BMI1 expression levels on the stoichiometry of the other members of the complex, or of the proteome bound to BMI1. Additionally, whether the biological functions that are regulated by BMI1 are entirely mediated by the PRC1 complex in GBM is also unknown.

Here, we have used a proteomics strategy based on mass spectrometry analysis of immuno-precipitated protein complexes and chromatin as well as expression modulation using CRISPR/dCAS9 to decipher the composition of PRC1 complexes and to characterize the BMI1 interactome in GBM. Impact on gene expression and key functional properties were then assessed in patient-derived GIC lines to advance our understanding of the mechanisms mediating BMI1 function in GBM.

## MATERIALS AND METHODS

### Cell culture

GBM cell lines U87MG and LN428 were grown in DMEM, high glucose with Glutamax (31966 Gibco, Life technologies) supplemented with 40 ml of FBS (Gibco, Life technologies) and 10 ml of Pen-Strep (Gibco 15140–122). Authentication for both cell lines was performed by Eurofins (Supplementary Data 10 and 11). Primary GIC cultures were carried out as described in supplementary experimental procedures. IDH wild-type status of these cell lines was determined by inspection of the DepMap portal by Broad Institute (https://depmap.org/portal/).

### Chromatin immunoprecipitation

ChIP was performed as previously described (43), with modifications as follows. Cells were fixed with an initial cross-linking step of 45 min with 2 mM Di(N-succinimidyl) glutarate (Sigma-Aldrich Cat. 80424) in 1× PBS (Sigma) at room temperature, followed by a 1× PBS (Sigma) wash and a second fixation step of 12 min with 1% formaldehyde (Sigma) in 1× PBS. After quenching with glycine, washes and lysis as described in (43), chromatin was sonicated using a Bioruptor Pico from Diagenode, on a 30 s on/off cycle for 20 cycles. Immunoprecipitation was performed using 200 μg of chromatin and 14 μg of antibodies BMI1 (39993, Actif Motif), RYBP (AB3637, Millipore) or H2AK119ub (Millipore 05–678). We did not use proteinase K removal step at any point or elute chromatin–protein complexes from protein G (Sigma) beads. We substituted these steps with 100 mM AMBIC (Sigma) washes of 30 min twice to prepare chromatin pull-downs for MS as described previously (44). Final chromatin-bound beads were resuspended in 40 μl of 100 mM AMBIC buffer for MS analysis.

### Protein immunoprecipitation

Immunoprecipitation was performed using 200 μg of protein and using Pierce MS-Compatible Magnetic IP Kit, protein A/G (90409, Thermo Fisher Scientific) following manufacturer instructions in SDS-free conditions. Protein extracts were pulled down using 10 μg BMI1 antibody (39993, Actif Motif) or 10 μg RYBP antibody (AB3637, Millipore). Protein eluates diluted in 40 μl of 100 mM AMBIC buffer (Sigma) were analysed with MS. Extensively validated antibodies were used (45–48). For IP experiments, unbound samples were analysed to confirm sensitivity of the beads. For MS experiments proteins unspecifically binding to beads were subtracted by comparing BMI1-IP samples with mock-IP samples without antibody for each condition.

### Mass spectrometry

Two independent cultures of two different GBM cell lines were analysed. Proteomic experiments were performed in two technical replicates per culture using mass spectrometry as reported (49). For ChIP samples, ChIP protein complex beads were digested into peptides using trypsin. In the case of IP samples, proteins were eluted from beads prior digestion with trypsin. Peptides were desalted using C18^+^carbon top tips (Glygen Corporation, TT2MC18.96) and eluted with 70% acetonitrile (ACN) with 0.1% formic acid. After drying in a speed-vac to remove ACN, dried peptides were dissolved in 0.1% TFA and analyzed by Nanoflow ultimate 3000 RSL nano instrument coupled on-line to a Q Exactive plus mass spectrometer (Thermo Fisher Scientific). Gradient elution was from 3% to 35% buffer B in 120 min at a flow rate 250 nl/min with buffer A being used to balance the mobile phase (buffer A was 0.1% formic acid in water and B was 0.1% formic acid in ACN). The mass spectrometer was controlled by Xcalibur software (version 4.0) and operated in the positive mode. The spray voltage was 1.95 kV and the capillary temperature was set to 255°C. The Q-Exactive plus was operated in data-dependent mode with one survey MS scan followed by 15 MS/MS scans. The full scans were acquired in the mass analyser at 375–1500 *m/z* with the resolution of 70 000, and the MS/MS scans were obtained with a resolution of 17 500.

### Proteomics bioinformatics

MS raw files were converted into Mascot Generic Format using Mascot Distiller (version 2.5.1) and searched against the SwissProt database restricted to human entries using the Mascot search daemon (version 2.5.0) with a FDR of ∼1% and restricted to the human entries. Allowed mass windows were 10 ppm and 25 mmu for parent and fragment mass to charge values, respectively. Variable modifications included in searches were oxidation of methionine, pyro-glu (N-term) and phosphorylation of serine, threonine and tyrosine. The mascot result (DAT) files were extracted into excel files for further normalization and statistical analysis. We removed proteins detected in mock samples where no antibody was used as pull-down to remove unspecific binding of proteins to the beads. Then, we calculated the fold-change of MS2 spectra intensity of BMI1-bound proteins comparing LN428^iCRBMI1^ and U87MG^aCRBMI1^ over their matching empty backbones LN428^iCRempty^ and U87MG^aCRempty^, respectively. Protein contaminants from MS list were removed using CRAPome software (CRAPome.org) (50). The mass spectrometry proteomics data have been deposited to the ProteomeXchange Consortium via the PRIDE partner repository with the dataset identifier PXD022057 and 10.6019/PXD022057

### Proximity ligation assay (PLA)

Assay was carried out using Duolink PLA Flow Cytometry assays (Merck, Sigma, DUO94002 and DUO94001) following manufacturer’s instructions. Briefly, 1 × 10^6^ cells were fixed in cold 4% PFA (Sigma) in 1× PBS (Sigma) for 20 min. Cells were spun at 1000 *g* for 5 min and pellets were washed twice with 1× PBS. Cell pellets were blocked with Duolink blocking solution at 37°C for 1 h. After that, cells were incubated with respective primary antibodies in 1:100 dilution [CBX8 (sc-374332, Santa Cruz Biotechnology), RYBP antibody (AB3637, Millipore), BMI1 (PLA0208, Sigma) and H3K27me3 (ab195477, Abcam)] with respective no antibody controls at 4°C o/n followed up by an incubation of secondary Duolink plus and minus antibodies (Duolink PLA anti mouse plus DUO92001 and duolink PLA anti rabbit minus DUO92005) in a 1:5 proportion at 37°C for 1 h. Ligation of antibodies was performed adding Duolink ligase in 1× ligation solution at a 1:40 dilution and incubated at 37°C for 1 h. Amplification of ligated products was performed using Duolink polymerases in 1× amplification buffer at a 1:80 dilution and incubated at 37°C o/n. Detection of amplified products was performed adding 1× detection buffer and incubating at 37°C for 1 h. Cells were washed with Duolink wash buffer twice and resuspended in 300 μl of 1× PBS followed by cell sorting of Green or FarRed positive cells using BD FACS Canto II Analyzer and replicates were analysed with FlowJo 10 software. Experiments were performed in three biological replicates (*n* = 3).

### Cholesterol cell viability assay

The number of metabolically active cells was measured using the CellTiter-Glo luminescent assay following manufacturer’s instructions (Promega). To assess effect of cholesterol production in cell viability, simvastatin (Sigma) at a concentration of 80 ng/ml was used as a drug to inhibit cholesterol biosynthesis, U-18666A (Abcam) at a concentration of 2.5 μM was used as an inhibitor of cholesterol transporter proteins and PTC 209 as a BMI1 inhibitor at a concentration of 5 μM (Sigma). Luminiscence was measured in plate reader (CLARIOstar BMG labtech, analysis software CLARIOstar MARS). Experiments were performed in three biological replicates (*n* = 3).

### Cell aggregation assay

Primary GBM cells were plated at 100% cell confluence in 96-well plates (Corning) coated with 10 μg/ml laminin (Sigma). We also added a red dye (Invitrogen, CellTracker Deep Red C34565) at a concentration of 5 μM as a cell tracker. Mitomycin C (Sigma) at 10 μg/ml concentration was added to the media for 40 min to stop cell proliferation. After mitomycin incubation, new media were added, and cells were imaged every 24 h for 7 days using IN Cell Analyzer 2000 cell imaging system (GE Healthcare). Cell aggregation was calculated as area covered by cells over empty surface and measured using IN CELL developer toolbox 1.9.2 software. Experiments were performed in three biological replicates (*n* = 3).

### RNA-sequencing

Total RNA was isolated from cell pellets from two indepedent cultures of each GIC line and condition using RNeasy Micro Purification Kit (Qiagen). RNA was digested with DNaseI (Applied Biosystem) in column following manufacturer’s instructions. Total RNA was sequenced using HiSeq platform with paired-end 150 bp (PE-150), 20 M reads at Novogene, Cambridge, UK. After assessing read quality and the potential presence of adapters via FastQC and TrimGalore, respectively (www.bioinformatrics.babraham.ac.uk), read mapping to Ensembl GRCh38 reference genome was performed using STAR v. 2.7.0 (51) with default parameters. R v. 3.5.1. was used to perform the rest of the RNA-Sequencing analysis. Bioconductor packages NOISeq (52), biomaRt (53) and edgeR were used to filter the data, annotate the genes and perform the differential expression analysis including TMM normalization, respectively (54). Specifically, lowly expressed genes (TPM < 1) and genes associated with Mt-RNA and rRNA were filtered out. After TMM normalization, a quasi-likelihood negative binomial generalized log-linear model (glmQLFit) was fitted to the read counts and dysregulated genes were considered significant at a *P*-value < 0.05. Alternative mRNA splicing was analyzed using the Bioconductor package DEXseq 1.28.3 as described previously (55). DEUs with Benjamini–Hochberg adjusted *P* < 0.05 were considered significant. Datasets have been deposited in Geo (GSE159747).

### Gene ontology, networks, functional analysis and GBM databases

Connectivity networks and functional analysis were obtained using STRING 10.5 (56) (string-db.org) and Ingenuity Pathway analysis (IPA, www.ingenuity.com) software packages. RNA expression levels of specific targets were assessed using GlioVis (57) (http://gliovis.bioinfo.cnio.es/). Profile plots for histone marks were created using GSMplots (58) and accession numbers from the GEO database.

## RESULTS

### Characterization of the BMI1 and RYBP interactome in GBM identifies canonical and noncanonical PRC1 protein networks

We first set out to identify GBM lines that could serve as adequate models to assess the BMI1 and RYBP interactome. BMI1 expression levels were assessed in a collection of GBM cell lines as compared to a commercially available iPSC-derived neural stem cell (iNSC) line. BMI1 expression was variable with most lines showing equal or increased expression as compared to iNSC (Supplementary Figure S1A). Interrogation of a publicly available collection of 48 patient-derived IDH-wild type glioblastoma initiating cells (GIC) lines (HGCC (59)) revealed equally variable expression levels between the cultures (Supplementary Figure S1B). We chose two IDH-wild-type GBM cell lines (U87MG and LN428) (60) and two GIC lines (U3118 and U3082) with levels of BMI1 overexpression mirroring the variation observed in GBM tumour samples (39,61,62). BMI1 overexpression was confirmed at protein level, as compared to adult brain; these lines expressed BMI1 at similar levels to foetal NSC and to a patient-derived medulloblastoma line (ICb1299), known to overexpress and be functionally dependent on BMI1 (63) (Supplementary Figure S1C).

To identify proteins interacting with BMI in GBM cells, immunoprecipitation for BMI1 was carried out followed by mass spectrometry (IP-MS) in U87MG and LN428 cell lines. We used cell lines as they could be sufficiently expanded to obtain enough protein for IP-MS while retaining similar BMI1 expression levels to patient-derived GIC lines. BMI1-specific IP was confirmed by western blotting and MS (Supplementary Figure S1D).

**Figure 1.**
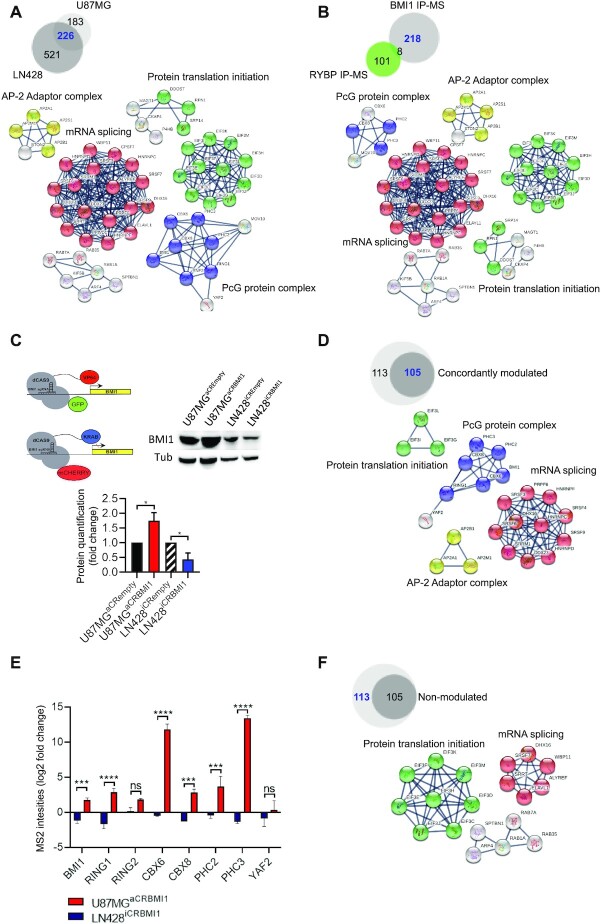
Characterization of the BMI1 interactome in GBM. (**A**) Venn diagram indicating BMI1 interactors in GBM cell lines (blue) and protein networks identified from these proteins, including PcG protein complex, protein translation initiation and elongation, mRNA splicing and AP-2 adaptor complex. (**B**) Venn diagram indicating BMI1 interactors not shared with RYBP (blue) and protein networks identified from these proteins. (**C**) Schematic illustrating CRISPR/dCAS9 plasmids to modulate BMI1 expression and western blot with quantification demonstrating BMI1 protein modulation (*n* = 3; one-way ANOVA, *P< 0.05). (**D**) Venn diagram showing BMI1 bound proteins, which are also concordantly modulated (blue) and their networks. (**E**) Bar plot showing PRC1 complex members concordantly modulated with BMI1. Fold change indicates different MS2 binding intensities obtained by calculating Log2 fold change of average MS2 spectra values of different replicates of LN428^iCRBMI1^ and U87MG^aCRBMI1^ over their matching empty backbones LN428^iCRempty^ and U87MG^aCRempty^ respectively (*n* = 2; two-way ANOVA, *P< 0.05, **P< 0.01, ***P< 0.001, ****P< 0.0001). (**F**) Venn diagram showing BMI1 bound proteins, which are not concordantly modulated (blue) and their networks.

A total of 747 and 409 BMI1-interacting proteins were detected in LN428 and U87MG, respectively (Figure 1A). Of these, 226 proteins were shared between the two cell lines, with 521 and 183 proteins identified only in LN428 and U87MG, respectively. We focused our attention on the 226 shared proteins as this group are most likely disease specific rather than cell line specific and independent of the degree of BMI1 overexpression. Analysis of protein association networks using String software and Ingenuity pathway analysis (IPA) indicated that BMI1-associated proteins mainly belong to four networks, namely PRC1 complex network (BMI1, RING1, CBX6, CBX8, RNF2, PHC2, PHC3, YAF2), mRNA splicing network, protein translation initiation network and proteins related to the AP-2 adaptor complex (AP2B1, AP2M1, AP2S1, AP2A1) (Figure 1A) (Supplementary Data S1 and S3). While the PRC1 complex network was expected, the AP-2 adaptor complex, mRNA splicing and protein translation initiation networks have not been previously linked to PRC1 genes. Taking advantage of publicly available datasets where RING1B, the catalytic subunit of the PRC1 complex was used as a pull-down protein in mouse embryonic stem cells (mESC) and neural progenitor cells (mNPCs) (64), we identified 8/226 and 19/226 shared proteins between our GBM BMI1 interactome and the Ring1B interactome in mESC and mNPC, respectively, (Supplementary Figure S1E and Supplementary Data 4) with PRC1 complex (BMI1, PHC2, PHC3, CBX8, YAF2 and RING1) as the main network enriched for in the shared proteins (Supplementary Figure S1E), raising the possibility that only a small proportion of PRC1 interactors are common between GBM cells and normal cells with stem cell properties.

Because BMI1 is a component of both cPRC1 and ncPRC1 complexes (20,21), we selected RYBP, a well-characterized member of ncPRC1 (65), as a pull-down target for our IP-MS studies on the same GBM cell lines to characterize the RYBP interactome and the composition of the ncPRC1. From this analysis, 294 and 339 RYBP interacting proteins were identified in U87MG and LN428 respectively (Supplementary Figure S1F). Of these, 109 proteins were shared between the two cell lines with 230 and 185 proteins identified in LN428 and U87MG (Supplementary Figure S1F). Again, we focused on the proteins shared between the two cell lines and used the String and IPA platforms to identify significantly enriched protein networks associated with RYBP. The ncPRC1 complex (RING1, RNF2 (RING2), BMI1, RYBP, WDR5, PCGF6 and FBRS) was identified as one of the protein networks, as expected, and RNA polymerase III complex (POLR1C POLR1D, POLR3B, POLR3C POLR3D, POLR3F, POLR3G, POLR3H, POLR3K and CRCP) was also enriched for (Supplementary Figure S1F, Supplementary Data 2 and 3). The latter has been linked to PcG genes via EZH2 interaction with the TFIIIC transcriptional factor complex at the promoter of Pol III regulated genes, which leads to their repression (66), but not to PRC1.

Next, we set out to dissect the composition of the cPRC1 versus the ncPRC1 in GBM lines by comparing the BMI1 (cPRC1 and ncPRC1) and the RYBP interactomes (ncPRC1) (Figure 1B). PHC2, PHC3, CBX6 and CBX8 were PcG proteins bound to BMI1 but not RYBP, in keeping with cPRC1, while WDR5, a component of ncPRC1 complexes, is bound to RYBP but not BMI1. Among the eight proteins shared between the BMI1 and RYBP proteome, BMI1, RING1 and RING2 were confirmed as belonging to both cPRC1 and ncPRC1 complexes. KCTD3, LS14A, SHKB1, KI67 and SPB1 were also identified as interacting with BMI1 and RYBP (Supplementary Data S3). Interestingly, all these proteins were upregulated in IDH-wild type GBM versus non-tumour samples upon inspection of the TCGA database (57), with all but KCTD3 showing significant upregulation and the latter a trend (Figure S1G).

All other protein networks (mRNA splicing network, protein translation initiation network and proteins related to the AP-2 adaptor complex) were enriched for in the BMI1 but not in the RYPB interactome, in keeping with these being linked to BMI1 in an ncPRC1-independent fashion (Figure 1B). RNA Pol III continued to be enriched for in the RYBP interactome, raising the possibility that this network may be linked to RYBP in a cPRC1-independent fashion (Supplementary Figure S1H).

This analysis provides a first characterization of the cPRC1 and ncPRC1 composition in GBM and identifies networks involved in biological processes not yet linked to the BMI1 interactome.

### Modulation of BMI1 expression levels affects the stoichiometry of other PRC1 components

Next, we set out to assess whether modulation of BMI1 expression would affect the BMI1 interactome. CRISPR/dCAS9 gene expression modulation system was used to increase and decrease *BMI1* expression in U87MG and LN428 cell lines respectively (Figure 1C). To this end, a GFP tagged system where a dCAS9 is fused to the transcriptional activation domain (VP64) (67) was used for activation (aCRISPR) and a mCHERRY tagged system where a dCAS9 is fused to the transcriptional inactivation domain (KRAB) (68) for inactivation (iCRISPR) and stable U87MG^aCRISPR^ and LN428^iCRISPR^ lines were generated (Figure 1C). BFP-tagged vectors containing a selection of short-guide RNA sequences targeting *BMI1* (sg-BMI1) were transduced into U87MG^aCRISPR^ and LN428^iCRISPR^ lines; BMI1 protein levels were assessed to select a suitable BMI1 sg-RNA for further studies. Sg-BMI1–3 was chosen to increase BMI1 transcription and protein levels, here named U87MG^aCRBMI1^ (Figure 1C and Supplementary Figure S1I) and sg-BMI1–5 to reduce BMI1 transcription and protein levels, here named LN428^iCRBMI1^ (Figure 1C and Supplementary Figure S1K). Cell proliferation assays confirmed reduced and increased proliferation in LN428^iCRBMI1^ and U87MG^aCRBMI1^ respectively, as expected (30,69–71) (Supplementary Figure S1J,L). IP-MS performed on the BMI1-modulated cell lines (U87MG^aCRBMI1^ and LN428^iCRBMI1^) identified 105/218 proteins of the BMI1 interactome as being concordantly modulated with BMI1 expression, namely increased in U87MG^aCRBMI1^ and decreased in LN428^iCRBMI1^ (Figure 1D, Supplementary Data S3). As expected, other members of the PRC1 complex—CBX6, CBX8, PHC2, PHC3, RING1, RING2, YAF2—were identified amongst the 105 proteins, thus indicating that BMI1 levels influence the stoichiometry of other structural PRC1 subunits (Figure 1E). BMI1 protein abundance detected by MS confirmed modulation of BMI1 protein levels by CRISPR/dCAS9 protein fusions system and validated our analytical approach (Figure 1E). The mRNA splicing network and proteins related to the AP-2 adaptor complex signalling network continued to be enriched in this subgroup, while protein translation initiation network was predominantly lost when modulation of BMI1 expression levels is considered (Figure 1D). Conversely, BMI1 expression levels did not affect abundance of 113/218 proteins of the BMI1 proteome. String analysis confirmed that most members of the protein translation initiation network and some members of the mRNA splicing network were independent of BMI1 expression levels (Figure 1F). Comparative analysis with the Ring1B interactome in mESC and mNPC revealed that a similar proportion of the proteins shared with our BMI1 interactome (9/19 and 4/8, respectively) were among those with and without abundancy concomitantly altered upon BMI1 modulation (Supplementary Figure S1M).

Our results indicate that only a proportion of the BMI1 interactome is concordantly modulated with BMI1 expression levels.

### Compositional analysis of the BMI1, RYBP and H2AK119ub chromatome identifies putative PRC1-independent BMI1 interactors

**Figure 2.**
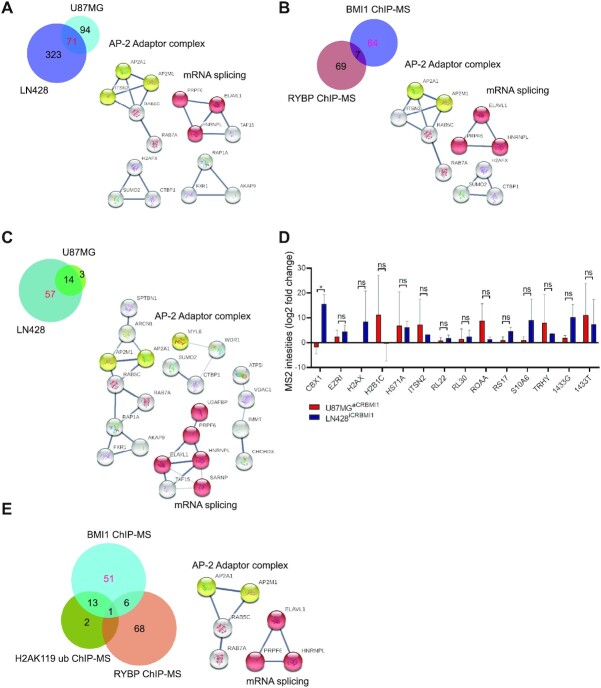
Characterisation of the BMI1 chromatome and comparative analysis with RYBP and H2AK119 chromatomes in GBM. (**A**) Venn diagram indicating chromatin-bound proteins shared between two GBM cell lines (pink) with associated networks, AP-2 adaptor complex (yellow) and mRNA splicing network (red). (**B**) Venn diagram identifying the BMI1 chromatome independently of RYBP (as proxy of ncPRC1) with associated networks, AP-2 adaptor complex (yellow) and mRNA splicing (red). (**C**) Venn diagram indicating proteins belonging to the BMI1 chromatome only (red) and related networks including the AP-2 adaptor complex (yellow) and mRNA splicing cluster (red). (**D**) Bar plot showing proteins found within the BMI1 and H2AK119ub chromatome and their abundancy upon BMI1 modulation. Fold change indicates different MS2 binding intensities obtained by calculating Log2 fold change of average MS2 spectra values of different replicates LN428^iCRBMI1^ and U87MG^aCRBMI1^ over their matching empty backbones LN428^iCRempty^ and U87MG^aCRempty^ respectively (*n* = 2; two-way ANOVA, ns, not significant, *P< 0.05). (**E**) Venn diagram indicating protein found only within the BMI1 chromatome (red) and related networks, AP-2 adaptor complex (yellow) and mRNA splicing (red).

Given the well characterized role of BMI1 in shaping chromatin structure, we set out to characterize the proteins associated to chromatin regions (chromatome) bound to BMI1 in the GBM cell lines, as compared to proteins interacting directly with BMI1, as assessed in the previous proteome screening. To this end, we used chromatin immunoprecipitation (ChIP) coupled with MS (ChIP-MS) with BMI1 as the bait protein. Furthermore, because BMI1 is part of both cPRC1 and ncPRC1, we performed ChIP-MS with RYBP, as a ncPRC1 component and the bait protein, thus allowing us to discriminate between the chromatome belonging to the cPRC1 and ncPRC1. Our results showed that 71 proteins of the BMI1 chromatome were shared between the two cell lines with AP-2 adaptor complex and mRNA splicing being identified as common networks (Figure 2A), similarly to our observations when analysing the BMI1 proteome (Figure 1B). Of the RYBP chromatome, 76 proteins were shared between the two cell lines (Supplementary Figure S2A). Strikingly, this analysis identified members of the PRC1 canonical complex such as CBX8, associated with RYBP in both GBM cell lines (Supplementary Figure S2A). Seven proteins (MYL6, AKAP9, ALDR, HS71A, KHDR1, ROA3, TAGL2) were part of both the BMI1 and RYBP chromatome (Figure 2B), four of which (AKAP9, MYL6, ALDR and TAGL2) were identified as upregulated in IDH-wild type GBM samples versus non-tumour using TCGA datasets (57) (Supplementary Figure S2B). Interestingly, 64 proteins out of 71 were part of the BMI1 but not the RYBP chromatome, with the AP-2 adaptor complex and mRNA splicing associated with BMI1 but not RYBP (Figure 2B). These analyses showed non-expected interactions between RYBP and the cPRC1 member CBX8, possibly due to proximity of different PRC1 complexes in the nucleosome. Furthermore, our results indicate that the AP-2 adaptor complex and mRNA group of proteins are associated with BMI1 as a cPRC1 but not ncPRC1 component.

The PRC1 complex catalyses ubiquitination of lysine 119 of histone H2A (H2AK119ub) via a RING1-mediated mechanism that is enhanced by BMI1. This epigenetic modification compacts chromatin and promotes gene silencing (18,72). Our results identified the interaction of BMI1 with proteins for which the biological functions are not yet known to be epigenetically regulated by the PRC1 complex. We used ChIP-MS for the histone mark H2AK119ub, representing the catalytic activity of PRC1 complex, to discriminate between enzymatic and non-enzymatic activities of the PRC1 complex. Seventeen proteins were found bound to H2AK119ub in both GBM cell lines (Supplementary Figure S2C). To elucidate which proteins belong to the BMI1 chromatome independently of the H2AK119ub chromatome, we comparatively analysed the two datasets and found 14 proteins common to BMI1 and H2AK119ub (PRC1) (Figure 2C). Among these 14 proteins, CBX1 abundance was concordantly impacted by BMI1 expression levels as modulated in the CRISPR edited GBM cell lines (Figure 2D). RL22, RL30 and RS17 are part of ribonucleoprotein complexes involved in translation initiation (73). Interestingly, RL30, RPS17, ROAA, S10A6 and TRHY were deregulated in IDH-wild type GBM versus non-tumour samples in the TCGA database (57) (Supplementary Figure S2D). 57 of the 71 proteins were identified within the BMI1 but not the H2AK119ub chromatome (Figure 2C), with mRNA splicing, which we also identified previously by IP-MS (Figure 1B), and the AP-2 adaptor complex being the networks most significantly enriched for (Figure 2C).

Next, to identify proteins associated to BMI1 independently of the PRC1 complex, we compared the three ChIP-MS datasets (BMI1, RYBP and H2AK119ub ChIP-MS) and found 51 proteins belonging to the BMI1 chromatome only, with mRNA splicing and the AP-2 adaptor complex continuing to be networks significantly enriched within this protein pool (Figure 2E and Supplementary Data 5), while two proteins were unique to the H2AK119ub chromatome (SPTBN4 and EMD), and 68 unique to the RYBP chromatome. Integration of all IP and ChIP-MS datasets identified 17 (1433E, AP2A1, AP2M1, CAPR1, EIFCL, ELAV1, PRP6, RL10, RL29, SPTB2, TBB5, TCPZ, U2AF1, FUBP3, FXR1, RAB7A, RRBP1) of the 51 proteins as shared between the BMI1 proteome and chromatome in GBM, none of which were found in the mNPC and only one (U2AF1) in the mESC Ring1B datasets (Supplementary Figure S2E).

Our results show that the BMI1-chromatome contained proteins also detected within the H2AK119ub chromatome, as expected. However, we also identified proteins unique to the BMI1-chromatome, that are not detected within the H2AK119ub or RYBP chromatomes, raising the possibility that they are independent of the catalytic activity of the PRC1, and possibly PRC1 independent.

### CBX8 is a component of the BMI1 interactome and RYBP chromatome in GBM cells and regulates essential tumour properties

**Figure 3.**
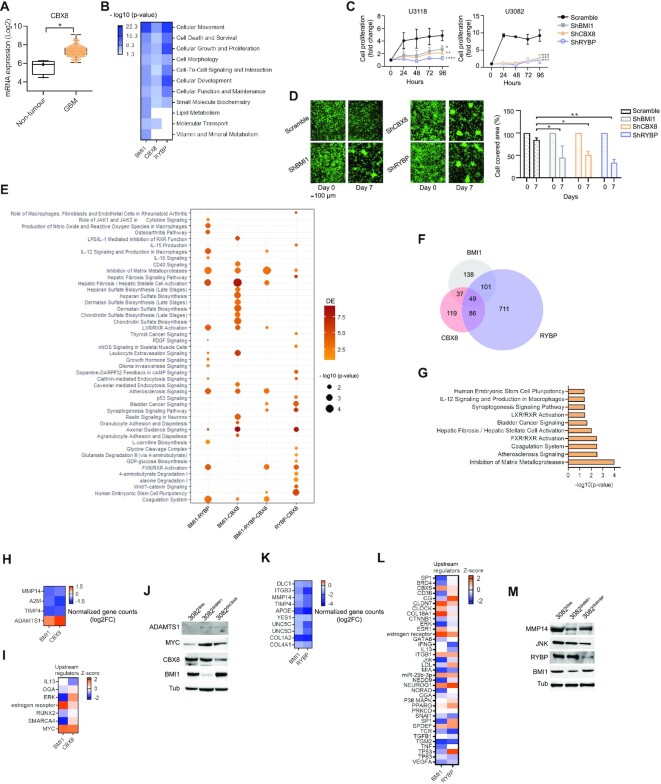
CBX8 regulates essential tumour properties in GBM. (**A**) Box plot indicating upregulation of CBX8 in GBM (*n* = 156) versus non-tumour samples (*n* = 4) in the TCGA database (Tukey's honest significant differences, *P< 0.05). (**B**) Enrichment score heatmap showing significantly impacted molecular functions (-log10 of *P*-value<0.05, *P*-score = or <1.3), as identified by IPA software, upon silencing of BMI1, CBX8 or RYBP. (**C**) Growth curve graphs showing decreased cell proliferation when BMI1, CBX8 or RYBP are silenced in two different GIC (U3118 and U3082) (*n* = 3; two-way ANOVA, *P<0.05, **P<0.01, ***P<0.001, ****P<0.0001). Fold change represents cell proliferation over seeded cells at 24, 48, 72 and 96 h time points. (**D**) Representative images (left) and quantification (right) of cell aggregation after 7 days upon silencing of BMI1, CBX8 or RYBP (*n* = 3; two-way ANOVA, *P< 0.05, **P< 0.01, ***P< 0.001, ****P< 0.0001); scale bar: 100 μm. (**E**) Dot plot of shared enriched canonical pathways dysregulated upon silencing BMI1, CBX8 or RYBP. Colour spectrum represents number of DE and circle size represent *P*-score values of significance of canonical pathways (*P*-value<0.05, *P*-score = or <1.3). (**F**) Venn diagram showing shared and exclusive DEG within the shBMI1, shCBX8 and shRYBP datasets. (**G**) Bar plot identifying inhibition of matrix metalloproteinases as the most enriched pathway upon analysis (IPA platform) of common DEG shared between shBMI1, shCBX8 and shRYBP datasets. (**H**) Log2 Fold change (Log2FC) of normalized gene counts heatmap showing differentially expressed genes belonging to inhibition of matrix metalloproteinase pathway. (**I**) Enrichment scores heatmap of predicted upstream regulators of the inhibition of matrix metalloproteinase pathway (- log10 of *P*-value<0.05, *P*-score = or <1.3), as DEG in shBMI1 and shCBX8 datasets. (**J**) Western blot for MYC, ADAMTS1 as well as BMI1 and CBX8 with TUBULIN as loading control (*n* = 2). (**K**) Log2 Fold change (Log2FC) of normalized gene counts heatmap showing differentially expressed genes belonging to inhibition of matrix metalloproteinase pathway. (**L**) Enrichment score heatmap of predicted upstream regulators as DEG in shBMI1 and shRYBP datasets (-log10 of *P*-value<0.05, *P*-score = or <1.3). (**M**) Western blot for JNK and MMP14 as well as BMI1 and RYBP are silenced. TUBULIN was used as loading control (*n* = 2).

cPRC1 and ncPRC1 complexes primarily differ in the presence of CBX members, which contribute to the recognition of the H3K27me3 mark catalysed by PRC2 (74,75). Our interactome screening identified CBX8 as part of the PRC1 complex in GBM; however, CBX8 was also found within the RYBP chromatome (Supplementary Figure S2A). Because CBX8 is significantly upregulated in GBM (Figure 3A) and may therefore play a role in GBM pathogenesis, we set out to confirm this interaction, and assess its functional role in GBM. Western blot analysis of BMI1-bound proteins upon immunoprecipitation confirmed the interaction between CBX8 and BMI1 in GBM cell lines and in patient-derived GIC lines (Supplementary Figure S3A, B). Next, proximity ligation assay (PLA) followed by FACS analysis was carried out to determine whether the CBX8–RYBP interaction at chromatome level could be confirmed. Detection of the interaction between CBX8 and H3K27me3 (Supplementary Figure S3C) was used as a positive control for the assay, as CBX8 has been described to recognize the H3K27me3 mark to tether the PRC1 complex to marked loci (76). We showed that CBX8 interacted with RYBP and BMI1 in GBM lines as well as in patient-derived GIC, as assessed by increased GFP intensity following the proximity of complementing GFP- tagged antibodies targeting CBX8 and RYBP (Supplementary Figure S3C,D). Whilst all cell line models demonstrated interaction of CBX8 with BMI1 and with RYBP, GIC showed higher GFP expression, possibly indicating more protein interactions between BMI1 and CBX8 or RYBP and CBX8 in GIC.

To begin to elucidate the contribution of CBX8 to the biological functions regulated by PRC1 in GBM pathogenesis, we silenced BMI1, CBX8 or RYBP in two GIC lines (U3118 and U3082) and compared their transcriptome. 325 (234 downregulated and 91 upregulated), 291 (195 downregulated and 96 upregulated) and 947 (399 downregulated and 548 upregulated) deregulated genes (DEG) were identified in shBMI1, shCBX8 and shRYBP, respectively (Supplementary Data S6 and 7). Gene Ontology enrichment analysis (IPA platform) was used to identify molecular functions shared between all conditions (Figure 3B). Cellular movement, cell survival, cell proliferation, cell morphology and cell-to-cell signalling interactions were the most significantly deregulated in all three comparisons (Figure 3B). Reduced cell proliferation was confirmed upon silencing of BMI1, CBX8 or RYBP in the two GIC lines (Figure 3C). Similarly, deregulation of cell adhesion and cell–cell interactions were confirmed in all three conditions in an assay whereby the degree of cell occupancy over plating surface was assessed and quantified between days 0 and 7 (Figure 3D). No impact on apoptotic cell death was observed, as assessed by measuring Caspase 3 activity (Supplementary Figure S3E).

Next, we set out to identify molecular pathways similarly impacted by silencing BMI1, CBX8 or RYBP. Deregulation of Axonal guidance signalling was noted, in keeping with previous observations of its epigenetic regulation during neurodevelopment (77) and GBM pathogenesis (78). Inhibition of metalloproteases stood out among those pathways dysregulated upon silencing of either of the three PRC1 components (Figure 3E), and this pathway was the most significantly deregulated when the 49 DEG shared between the three conditions were analysed (Figure 3F,G and Supplementary Data 7). MMP14, A2M and TIMP4 were downregulated and ADAMTS1 was upregulated in both shBMI1 and shCBX8 datasets (Figure 3H), in keeping with previous observations in mouse GIC and NPC (79). Interestingly, MYC, a predicted upstream regulator of ADAMTS1, was also upregulated in both shBMI1 and shCBX8 datasets (Figure 3I), a finding which was confirmed in an independent silencing experiment in the GIC line, U3082 (Figure 3J).

MMP14 as well as other extracellular matrix (ECM) genes were also identified as concordantly downregulated upon BMI1 and RYBP silencing (Figure 3K), again in keeping with previous observations in GIC and NPC (79). In this case though, JNK was predicted *in silico* to act as upstream regulator of MMP14 (Figure 3L) and downregulation of MMP14 and JNK were confirmed on an independently silenced GIC line, U3082 (Figure 3M). Dysregulated JNK pathway upon BMI1 knock-down has been previously shown in GSC and NPC (79) and in chronic myeloid leukaemia (CML) cell lines, where it led to upregulation of CCNG2, a G2 cyclin, which in turn increased phosphorylation of phosphate pathways, including the JNK pathway (80), raising the possibility that members of the metalloproteases signalling cascade could be regulated by ncPRC1 and JNK pathway in GBM.

Our results show that members of the cPRC1 and ncPRC1 regulate classical PRC1-mediated biological functions in GBM through different cellular cascades.

### mRNA splicing is regulated by BMI1 but not CBX8 in GBM

Next, we set out to understand the functional relevance of selected novel protein networks identified as enriched for in the BMI1 interactome.

The RNA splicing network is independent of canonical PRC1 enzymatic activity, and non-canonical PRC1 interactome and chromatome (Figure 2B and E). Among the network components, PRP6 was selected for validation and further analysis because it is a bridging factor between U5 and U4/U6 snRNPS and it participates in the assembly of the spliceosome machinery that regulates alternative splicing events (81). Moreover, PRP6 promotes tumorigenesis via the regulation of alternative splicing in multiple cancers (82). BMI1 IP followed by western blotting confirmed that PRP6 is bound to BMI1 in GBM cell lines as well as GIC (Supplementary Figure S4A, B), although it seemed to be not concordantly modulated by BMI1 expression levels in GIC (Figure 1D), possibly because of the difference in sensitivity between IP-MS and IP-western blot or because of the different silencing method used in GIC.

RNA-sequencing analysis on two GIC lines (U3118 and U3082) upon PRP6 silencing (Supplementary Figure S4C) identified 854 (500 downregulated and 354 upregulated) DEG (Supplementary Data S6 and 7). Analysis of the DEG on the IPA platform revealed enrichment for cellular movement, cell death and proliferation among the top biological functions affected upon PRP6 silencing, similar to our observations after BMI1 silencing (Supplementary Figure S4D). Reduced cell proliferation was confirmed upon silencing of PRP6 in both GIC lines (Supplementary Figure S4E), although no impact on cell movement or cell death were observed upon PRP6 silencing (Supplementary Figure S4F,G), indicating a different biological impact on some of the biological functions affected as compared to BMI1 silencing (Figure 3D).

**Figure 4.**
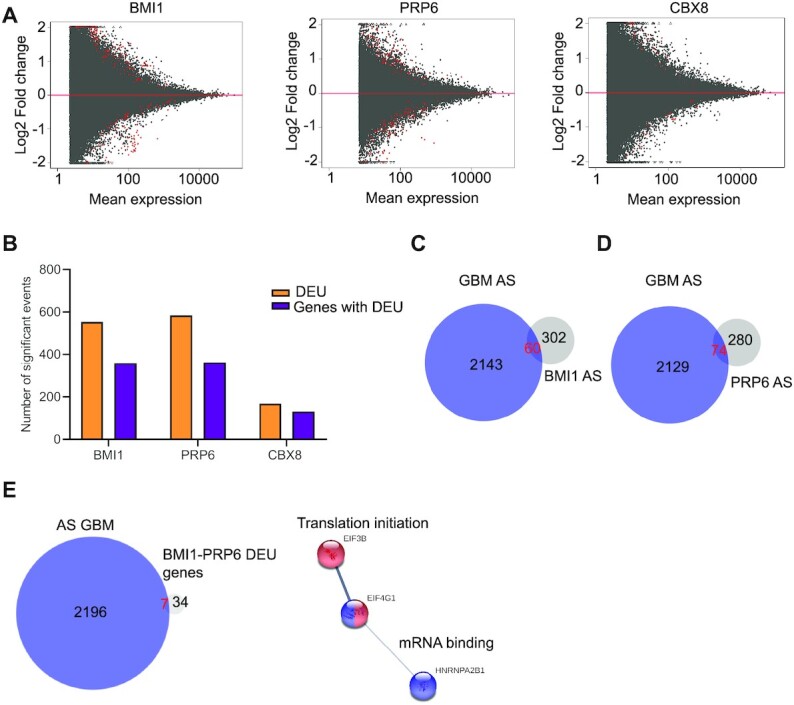
BMI1 regulates alternative splicing in GBM. (**A**) MA plots indicating Log2 fold change versus mean expression to show significant genes with DEU (Benjamini–Hochberg adjusted *P*< 0.05, coloured in red) upon BMI1, PRP6 and CBX8 silencing. (**B**) Histogram showing number of DEU events and genes with DEU undergoing alternative splicing upon BMI1, PRP6 and CBX8 silencing (Benjamini–Hochberg adjusted *P*< 0.05). (**C**) Venn diagram showing common DEU genes upon BMI1 silencing and previously identified prognostically relevant genes with alternative splicing in GBM (AS GBM). (**D**) Venn diagram showing common DEU genes upon PRP6 silencing and previously identified prognostically relevant AS-GBM genes. (**E**) Venn diagram showing common and exclusive genes with DEU upon BMI1 and PRP6 silencing and previously identified prognostically relevant AS GBM genes with translation initiation (red) and mRNA binding (blue) as networks enriched for.

To assess a potential role of BMI1 and PRP6 in mRNA splicing, differential exon usage (DEU), which accounts for changes in the relative use of exons in genes caused by a defined experimental condition, was calculated in transcriptomic datasets upon silencing of BMI1, PRP6 or CBX8, the latter never linked to alternative splicing. We observed that, a significantly higher number of genes with DEU were identified upon silencing of BMI1 or PRP6 (Figure 4B), as shown by statistically significant changes in exon usage as compared to CBX8 (Fisher’s exact test *P* < 0.0001) (Figure 4A and Supplementary Data 8).

As genes subjected to alternative splicing have been shown to impact on GBM prognosis (83), we assessed whether the genes with DEU identified in our experimental conditions would overlap with those previously identified as prognostically relevant in GBM. 60/362 and 74/354 genes with DEU identified upon BMI1 or PRP6 silencing respectively, were also identified as belonging to alternatively spliced genes in GBM (83) (Figure 4C and D; Supplementary Data 9), an overlap which is not random (hypergeometric test *P* << 0.001). Networks including Translational Elongation and Cholesterol Biosynthesis are among those most enriched for upon BMI1 silencing (Supplementary Figure S4H and Supplementary Data S9), and Structural Constituent of ribosome, Translation initiation and Alternative Spicing among those most enriched for upon PRP6 silencing (Supplementary Figure S4I and Supplementary Data S9). 41 genes with DEU were common to conditions where BMI1 or PRP6 had been silenced with 7/41 belonging to genes previously associated with GBM prognosis (APLP2, EIF3B, EIF4G1, HNRNPA2B1, MACF1, DST, TXNRD2; Supplementary Data 9) (83), with translation initiation and mRNA binding being the network enriched for (Figure 4E and Supplementary data 9).

In summary, our data show higher number of genes with DEU upon BMI1 or PRP6 silencing as compared to silencing of CBX8, although only a minority of the affected genes are shared between the two conditions. A proportion of these differentially spliced genes are known to contribute to determining GBM prognosis, raising the possibility that BMI1 regulates mRNA splicing in GBM, although the precise mechanism and whether it is a direct or indirect effect remain to be determined.

### BMI1 modulates cholesterol transport in a PRC1-independent fashion

**Figure 5.**
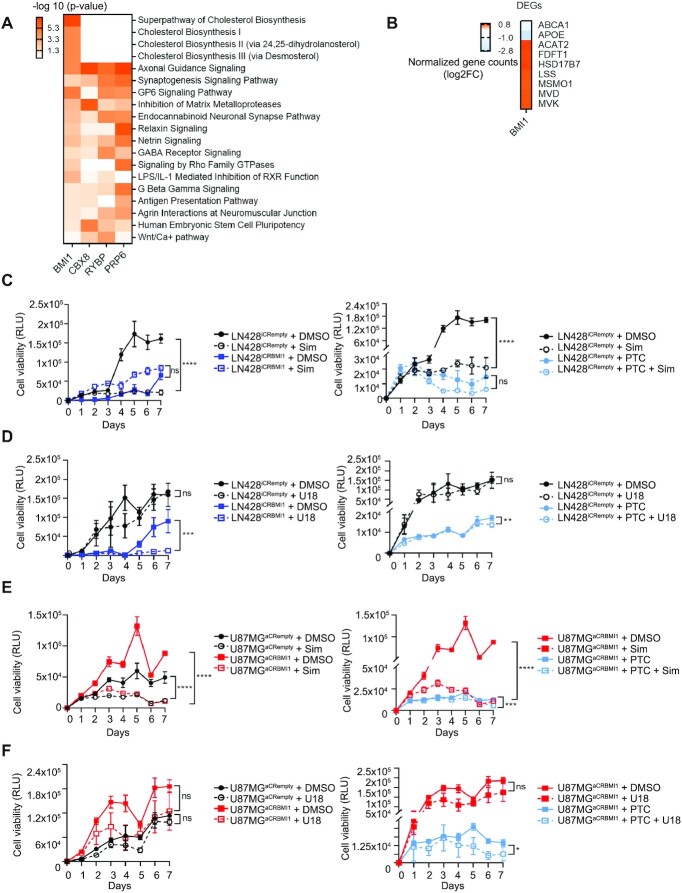
BMI1 regulates cholesterol metabolism in GBM. (**A**) Enrichment scores heatmap showing significant dysregulated molecular pathways (IPA software, - log10 of *P*-value < 0.05, *P*-score = or <1.3) upon BMI1, CBX8, RYBP or PRP6 silencing. (**B**) Log2 Fold change (Log2FC) of normalized gene counts heatmap showing differentially expressed genes (DEG) belonging to cholesterol metabolism (production and transport) in the shBMI1 dataset (IPA software, -log10 of *P*-value < 0.05, *P*-score = or <1.3). (**C**) Cell viability assay, assessed as relative luminescence units (RLU), in LN428^iCRempty^ versus LN428^iCRBMI1^ in the presence of 5 μM DMSO, 80 ng/ml Simvastatin (Sim) or 5 μM PTC 209 (PTC) and in the combination of 80 ng/ml Simvastatin (Sim) and 5 μM PTC 209 (PTC) (*n* = 3; two-way ANOVA, ns, not significant, *P< 0.05, **P< 0.01, ***P< 0.001, ****P< 0.0001). (**D**) Cell viability assay, assessed as relative luminescence units (RLU) in LN428^iCRempty^ versus LN428^iCRBMI1^ in the presence of 5 μM DMSO, 2.5 mM U1866A (U18) or 5 μM PTC 209 (PTC) and in the combination of 2.5 mM U1866A (U18) and 5 μM PTC 209 (PTC) (*n* = 3, two-way ANOVA, ns, not significant, *P< 0.05, **P< 0.01, ***P< 0.001, ****P< 0.0001). (**E**) Cell viability assay, as assessed by relative luminescence units (RLU) in LN428^aCRempty^ versus LN428^aCRBMI1^ in the presence of 5 μM DMSO, 80 ng/ml Simvastatin (Sim) or 5 μM PTC 209 (PTC) and the combination of 80 ng/ml Simvastatin (Sim) and 5 μM PTC 209 (PTC) (*n* = 3, two-way ANOVA, ns, not significant, *P< 0.05, **P< 0.01, ***P< 0.001, ****P< 0.0001). (**F**) Cell viability assay (relative luminescence units, RLU) in LN428^aCRempty^ versus LN428^aCRBMI1^ in the presence of 5 μM DMSO, 2.5 mM U1866A (U18) or 5 μM PTC 209 (PTC) and the combination of 2.5 mM U1866A (U18) and 5 μM PTC 209 (PTC) respectively (*n* = 3, two-way ANOVA, ns, not significant, *P< 0.05, **P< 0.01, ***P< 0.001, ****P< 0.0001).

To gain further insight into the biological function of these novel BMI1 interactors, we performed comparative analysis of the transcriptome of GIC primary lines upon silencing of BMI1, RYBP, CBX8 or PRP6. This analysis highlighted a series of pathways regulating cholesterol metabolism exclusively upon silencing of BMI1 (Figure 5A and Supplementary Figure S5A). After closer inspection of the transcriptomic data obtained after BMI1 silencing, we observed upregulation of the expression of enzymes involved in different steps of the cholesterol synthesis pathway (ACAT2, FDFT1, HSD17B7, LSS, MSM01, MVD and MVK genes) (Figure 5B and Supplementary Figure S5B), as well as downregulation of cholesterol membrane transporter genes (ABCA1 and APOE) (Figure 5B). Because none of these proteins were identified as binding BMI1 directly in our interactome screening and they did not show enrichment for BMI1 binding in ChIP-Seq datasets (37), it is conceivable that BMI1 regulates their expression, and therefore cholesterol metabolism in an indirect fashion. Interestingly, we identified FDFT1, a gene encoding the first specific enzyme in the mevalonate pathway for cholesterol biosynthesis (84), among those deregulated with DEU in exon E050 upon BMI1 silencing (Supplementary Figure S5C and Supplementary Data S9). Furthermore, although not a DEG in our datasets, we identified HMGCS1, an HMG-CoA synthase that catalyses conversion of acetate to mevalonate in cholesterol synthesis (85), as displaying DEU in exon E021 upon BMI1 silencing (Supplementary Figure S5C).

To understand the functional relevance of these findings, we used drugs interfering with cholesterol biosynthesis and transport in the context of BMI1 expression modulation and tested their impact on cell viability. GBM cell lines (U87MG and LN428) were treated with simvastatin, a drug that inhibits cholesterol biosynthesis and U18666A, a cholesterol transport inhibitor. We show that while simvastatin significantly impairs cell viability, no effect was exerted by U18666A in both unedited lines (Figure 5C and D) and no synergistic or additive effect was observed when both drugs were combined (Supplementary Figure S5D). Upon BMI1 silencing (LN428^iCRBMI1^), no additional effect was observed upon simvastatin treatment at the dose tested (Figure 5C). Pharmacological inhibition of BMI1 with PTC 209 confirmed a similar effect to simvastatin, as no significant further reduction of viability was observed (Figure 5C). Conversely, treatment with U1866A negatively impacted cell viability only in the context of BMI1 silencing, either upon editing (LN428^iCRBMI1^) or drug induced (PTC 209 treated) (Figure 5D).

On examination of GBM cells where CRISPR-mediated BMI1 overexpression was engineered, simvastatin was found to be equally effective in impairing cell viability independently of BMI1 expression levels (Figure 5E), while U1866A did elicit a negative impact on cell viability only upon pharmacological inhibition of BMI1, but not when BMI1 expression levels were increased (Figure 5F). No synergistic or additive effect was observed when both drugs were combined (Supplementary Figure S5E).

Our results raise the possibility that BMI1 supports cell viability in GBM by enhancing cholesterol metabolism, synthesis and transport at the cell membrane.

## DISCUSSION

To delineate protein binding partners of BMI1 and expand the current understanding of its biological functions in GBM, we used an IP-MS approach to comprehensively characterize BMI1 protein–protein interactions in GBM lines. We focused on BMI1 because of the growing body of evidence indicating that specific PCGF proteins confer cell type–specific non-overlapping functions to PRC1 complexes (13), with BMI1 (PCGF4) being required for proliferation and self-renewal of NSC (86) while at the same time having a well-documented role in GBM and being associated with a poor prognosis in cancer (20,32,87–90).

We show that CBX6, CBX8, PHC2, PHC3, RING1 and RING2 are the members of the PRC1 complex bound to BMI1 in GBM lines. Furthermore, interaction networks constructed with BMI1-bound proteins also highlighted the AP-2 adaptor complex, mRNA splicing and protein translation initiation networks, which have not been previously linked to PcG genes. Because complementary analysis of the BMI1 chromatome showed AP-2 adaptor complex and mRNA splicing as enriched for independently of the H2AK119ub chromatome, it is conceivable these biological functions may be regulated by BMI1 independently of the catalytic activity of PRC1 in GBM. Comparison with publicly available datasets of IP-MS using Ring1B as pull down antibody in murine NPC (64) revealed a 11.5% overlap with our dataset, mainly comprising PRC1 complex members. Because PRC1 complex composition is highly conserved within the same cell type across mammals (91), these data suggest that both similarities and differences between GBM cells and a progenitor cell, which have been shown to act as cell of origin of at least a proportion of these neoplasms (9) could apply to the human context. The modest overlap with the proteins previously identified as bound to Ring1B in NPC suggests that these proteins and networks may be specifically impacted in GBM.

Overexpression of PRC1 components in cancer may influence its interacting partners and shift PRC1 homeostasis. We show that most of PRC1 members detected in the GBM interactome are stoichiometrically regulated upon modulation of BMI1 expression via CRISPR/CAS9 protein fusions with a transcriptional activation domain (VP64 (67,92)) or a transcriptional inactivation domain (Krüppel-associated box (93)).

Interestingly, we identified CBX8 within the RYBP chromatome although not directly bound to RYBP, raising the possibility that cPRC1 and ncPRC1 complexes work in proximity in GBM. CBX8 promotes cell growth in multiple cancers including breast cancer, leukaemia, oesophageal carcinoma, colorectal and HCC (87,94–96). However, little is known about its function in GBM, with only one study showing overexpression of CBX8 in GBM cells as compared to astrocytes (97). It is conceivable that the increase in CBX8 proteins within the cPRC1 complex could lead to enhanced binding to H3K37me3-marked loci and promote their silencing, leading to transcriptional repression. However, it remains unclear how members of the complexes relate to transcriptional and biological functions. Here we show that CBX8 regulates cell proliferation and cell aggregation in GIC similarly to BMI1 and RYBP.

Interestingly among dysregulated pathways common to PRC1 components (BMI1, RYBP and CBX8), inhibition of metalloproteases stood out as the most significantly dysregulated molecular pathway, when DEG were compared after silencing of either of the three PRC1 components. Among the impacted genes, those shared between shBMI1 and shCBX8 datasets were predicted to be regulated by MYC, and those shared between the shBMI1 and shRYBP datasets by JNK. ADAMTS1 (A Disintegrin And Metalloproteinase with ThromboSpondin Motifs 1) is an extracellular protease involved in cell proliferation, angiogenesis and organogenesis (98). It is upregulated in GBM and it degrades brevican, one of the most abundant proteoglycans in adult brain, and is active in glioma cell invasion (99). Increased levels of ADAMTS1 lead to an increased cleaved IGFBP2, one of its target genes, which is associated with poor prognosis in gliomas (100). ECM genes including MMP (which promote the degradation of ECM and are involved in the regulation of cellular processes such as cell proliferation, differentiation, apoptosis and migration (101,102)) were DEG upon silencing of BMI1 or RYBP. Both ADAMTS and MMP family members are known to be BMI1 targets in GIC and NPC, as assessed by ChIP Seq (79), and their predicted upstream regulator, MYC, has also been described to be controlled by BMI1 in prostate cancer (103). Moreover, the phosphorylation of JNK is controlled by the cyclin CCNG2, which is a direct BMI1 target in myeloid leukaemia (80). Taken together these data provide an interpretative framework for how canonical and non-canonical PRC1 could converge on the modulation of key cellular properties either directly or indirectly. We show that BMI1 interacts with members of the spliceosome machinery, a regulatory mechanism characterized by extensive and dynamic protein interactions during tumour formation (104). Alternative splicing is regulated by interactions between RNA-binding proteins and specific pre-mRNA sequences with two main classes of RNA-binding proteins acting as splicing factors, serine-arginine (SR) proteins and heteronuclear riboproteins (hnRNP) (105). The role of BMI1 in alternative splicing has been described in epithelial-to-mesenchymal transition (EMT) (106), but not in brain tumours. We identified PRP6, a member of the small ribonucleoprotein (snRNP) spliceosome complex (107), within the BMI1-interactome. Missense mutations in this gene lead to incorrect pre-mRNA splicing in retinitis pigmentosa (108), while inhibition of the spliceosome machinery reduces cell proliferation of colon cancer cells (109). We show high number of genes with DEU upon silencing of BMI1 or PRP6, including genes involved in translation initiation as well as those encoding for ribonucleoproteins and SR pre-mRNA splicing proteins. Interestingly, SR proteins were identified within the BMI1-interactome, indicating that BMI1 may regulate alternative splicing events by direct interaction with splicing factors. Ribosomal mRNA can undergo alternative splicing events leading to different ribosomal proteins (110,111). We observed that upon PRP6 silencing a group of structural components of the ribosome displayed alternative splicing, thus indicating that PRP6 could regulate translation outcomes by modulating alternative splicing of ribosomal subunits.

Finally, we show that BMI1 plays a role in cholesterol metabolism. Cellular metabolism within the CNS is known to involve higher lipid contents compared with other systemic organs (112) with the majority of cholesterol being synthesized via *de novo* biosynthesis by astrocytes and delivered to neurons within high-density lipoproteins containing apolipoprotein E (113). Excess intracellular cholesterol is eliminated by promoting cholesterol efflux transporters such as ABCA1, which are regulated by LXR/RXR ligands in various systems (114), including GBM (115), and uptake is suppressed through degeneration of low-density protein receptor (LDLR) (116). The metabolic requirement of GBM cells is supplied mainly by exogenously synthesized cholesterol and intracellular cholesterol metabolism has become an attractive novel target in GBM (117,118), despite little knowledge about how it is regulated in this tumour. We show downregulation of the expression of lipid transporters (APOE apolipoprotein and the ABCA1 transporter) and deregulation of the FXR/RXR pathway upon BMI1 silencing in GIC, indicating a possible impairment of cellular cholesterol flux, in keeping with the notion that these cells rely on cholesterol transporters to maintain intracellular cholesterol levels.

Our observation of impairment of cell viability upon treatment with a cholesterol transport blocker only upon BMI1 silencing is in keeping with this interpretation. Upregulation of genes involved in the biosynthesis of cholesterol, without additive/synergistic effect upon treatment with a cholesterol biosynthesis inhibitor, would suggest this to be a compensatory mechanism to maintain cholesterol levels in the cell in the absence of an efficient cholesterol flux, as induced by BMI1 silencing. Interestingly, both well characterised and novel BMI1/PRC1 targets, such as Tp53 (119–121) and Estrogen Receptor (122), respectively, are predicted *in silico* to be upstream regulators of the lipid transporter genes deregulated upon BMI1 silencing. This raises the possibility that BMI1 may impact cholesterol metabolism via these intermediate regulators. Importantly, no enrichment for pathways involved in cholesterol metabolism was observed when either CBX8 or RYBP were silenced, and none of the proteins encoded by DEG upon BMI1 silencing and belonging to these pathways were identified in the BMI1 interactome or as being enriched for BMI1 binding in published ChIP-Seq studies in GBM (79). These results raise the possibility that cholesterol metabolism is regulated by BMI1 in a PRC1-independent fashion in GBM, although the possibility of a PRC1-dependent modulation of a yet uncharacterized intermediate regulator cannot be excluded. Interestingly, HMGCS1 and FDFT1, key genes of cholesterol biosynthesis, are among those genes with DEU upon BMI1 silencing. HMGCR and LDL have been shown to undergo alternative splicing events, resulting in a reduction of protein or enzymatic activity, in response to increased cellular sterol levels in hepatoma cell lines (123). Moreover, previous work has shown that upon overexpression of HNRPA1, a heterogeneous nuclear ribonoucleoprotein, there was a reduction of cholesterol synthesis by reducing HMGCR enzyme activity, resulting in increased LDL-C cholesterol uptake and an increase in expression in the cholesterol transporter APOB. (124). Our observation of an alternative splicing event in the exon 21 of the cholesterol reductase HMGCS1 (which condenses acetyl-CoA with acetoacetyl-CoA forming HMG-CoA, a substrate for HMGCR), together with the identification of the ribonucleoprotein HNRNPA2B1 as a gene with alternative splicing events in exons 15 and 27 upon silencing of BMI1 or PRP6, raise the possibility that BMI1 regulates cholesterol biosynthesis by modulating alternative splicing forms of genes involved in cholesterol metabolism.

In summary, by characterizing the BMI1 interactome we have identified novel regulatory roles for this protein in GBM, which will facilitate further exploration of its druggability in this currently untreatable, aggressive form of brain cancer.

## DATA AVAILABILITY

The mass spectrometry proteomics data have been deposited to the ProteomeXchange Consortium via the PRIDE partner repository with the dataset identifier PXD022057 and 10.6019/PXD022057. RNAseq datasets have been deposited in Geo (GSE159747).

## Supplementary Material

zcab009_Supplemental_Files
