# Correction to ‘Elucidation of the BMI1 interactome identifies novel regulatory roles in glioblastoma’

**DOI:** 10.1093/narcan/zcab020

**Published:** 2021-05-25

**Authors:** Verónica Freire-Benéitez, Nicola Pomella, Thomas O Millner, Anaëlle A Dumas, Maria Victoria Niklison-Chirou, Eleni Maniati, Jun Wang, Vinothini Rajeeve, Pedro Cutillas, Silvia Marino

**Affiliations:** Blizard Institute, Barts and the London School of Medicine and Dentistry, Queen Mary University of London, E1 2AT, London, UK; Blizard Institute, Barts and the London School of Medicine and Dentistry, Queen Mary University of London, E1 2AT, London, UK; Blizard Institute, Barts and the London School of Medicine and Dentistry, Queen Mary University of London, E1 2AT, London, UK; Blizard Institute, Barts and the London School of Medicine and Dentistry, Queen Mary University of London, E1 2AT, London, UK; Blizard Institute, Barts and the London School of Medicine and Dentistry, Queen Mary University of London, E1 2AT, London, UK; Centre for Therapeutic Innovation (CTI-Bath), Department of Pharmacy and Pharmacology, University of Bath, Bath BA2 7AY, UK; Barts Cancer Institute, Barts and the London School of Medicine and Dentistry, Queen Mary University of London, London EC1M 6AS, UK; Barts Cancer Institute, Barts and the London School of Medicine and Dentistry, Queen Mary University of London, London EC1M 6AS, UK; Barts Cancer Institute, Barts and the London School of Medicine and Dentistry, Queen Mary University of London, London EC1M 6AS, UK; Barts Cancer Institute, Barts and the London School of Medicine and Dentistry, Queen Mary University of London, London EC1M 6AS, UK; Blizard Institute, Barts and the London School of Medicine and Dentistry, Queen Mary University of London, E1 2AT, London, UK

*NAR Cancer*, Volume 3, Issue 1, March 2021, zcab009, https://doi.org/10.1093/narcan/zcab009

The Authors wish to add new sources of Funding (in bold):

