# Supplementary material for: Elucidation of the BMI1 interactome identifies novel regulatory roles in glioblastoma
Source: NAR Cancer. 2021 Mar 22;3(1):zcab009. doi: 10.1093/narcan/zcab009 (PMC8210184; doi:10.1093/narcan/zcab009)

SUPPLEMENTARY MATERIALS AND METHODS

Production of shRNA and CRISPR inactivation or activation lentiviral vectors and gene silencing.

We performed a first lentiviral infection of GBM cell lines U87MG and LN428 to create cell lines stably expressing dCAS9-VP64 and dCAS9-KRAB using previously described plasmids (Gilbert et al., 2014; Konermann et al., 2015) (Supplementary Table 1). We performed a second lentiviral infection using plasmids that contained short guide (sg) sequences targeting *BMI1*. To achieve this, we cloned, using as an enzyme BbsI (Thermo Fisher Scientific), 20-nucleotide sg targeting *BMI1* following a described CRISPR protocol (Ran et al., 2013) into a plasmid containing a U6 promoter to drive *sgBMI1* expression; *BFP* expression was used for FAC sorting as described before (Koike-Yusa et al., 2014). Sequences of sgRNAs targeting BMI1 are listed in Supplementary Table 2. GIPZ lentiviral vectors containing a short hairpin sequence targeting *BMI1*, *CBX8*, *RYBP and PRP6* and containing also *GFP* and puromycin gene sequences were purchased from Horizon (Dharmacon), UK (Supplementary Table 3) and used to transduce GBM primary cells (GIC) to achieve knock down of respective target genes. Viral packaging, production and titre determination were carried out as previously reported (Tang et al., 2015). Cells were transfected in two overnight rounds with 9.3×10^5 TU/ml for cell line and one overnight round with 93000 TU/ml for primary cells adding fresh media immediately after. After 96h, GFP or mCHERRY and BFP positive cells were single cell sorted using an Aria IIIu Cell sorter followed by puromicyn (Sigma) selection at a final concentration of 0.5 µg/ml for GSCs and 1 µg/ml for GBM cell lines. We assessed protein expression by Western blot. We performed a minimum of three biological replicates per knock down. We selected two biological replicates for MS analysis for GBM cell lines and two biological replicates for RNA-sequencing for GIC.

Primary cell culture conditions

Fetal striatum 15-week

Cells were plated into 6-well plates (Corning) coated with laminin (Sigma) at a final concentration of 6 mg/ml. Media was prepared mixing 500 ml DMEM/HAMS-F12 (Sigma 8437), 7.25 ml Glucose (Sigma G8644), 5 ml MEM NEAA 100x (Gibco 11140-035), 5 ml Pen-Strep (Gibco 15140-122), 800 uL BSA Solution 7.5% (Gibco 15260-037), 1 mL β-mercaptoethanol 50mM (Gibco 31350-010), 5 ml B27 Supplement 50x (LifeTech/Gibco 17504-044), 2.5ml N2 Supplement 100x (LifeTech/Gibco 17502-048). Before adding to the cells, complete media was supplemented with mouse EGF to final concentration 10 ng/ml (315-09, Peprotech), human FGF to final concentration 10 ng/ml (100-18b, Peprotech) and laminin (Sigma) to final concentration 1 ug/ml). Cells were split using accutase (Invitrogen).

ICb1299 primary medulloblastoma cells

Cell were grown in 100 mm dishes (Corning) in DMEM high glucose, Glutamax (Gibco 61965026) and supplemented with 40 ml of FBS (Gibco, Life technologies) and 10 ml of Pen-Strep (Gibco 15140-122). Cells were split using accutase (Invitrogen).

U3118 and U3082 GIC

Cells were obtained from The Human Glioblastoma cell culture resource (Xie et al., 2015) and cultured on Primaria 60 mm dish (Corning 353802) coated with laminin (Sigma) at a final concentration of 10µg/ml. Media was prepared as follows: Neurobasal (Gibco 21103-049) and DMEM/F12 glutamax (Gibco 31331-093) in 1:1 proportion, 5 ml of Pen-Strep (Gibco 15140-122), B27 50 X (12587-010,) and N2 100 X (17502-048). Before adding to the cells, media was supplemented with human EGF (Preprotech AF100-15) to final concentration 10ng/ml and human FGF-basic (Preprotech 100-18B) to final

Mycoplasma contamination screening was negative.

Proliferation and apoptosis assays

Proliferation of GBM cells transduced with CRISPR lentiviral constructs targeting BMI1 and GBM primary cells transduced with plasmids coding for shBMI1, shCBX8, shRYBP, and shPRP6 were counted using a plate reader (CLARIOstar BMG labtech, analysis software CLARIOstar MARS) following manufacturer's instructions. Apoptosis was assessed by measuring activity levels of Caspase 3(Invitrogen, 88-7004) following manufacturer's instructions and reading absorbance at 450 nm in plate reader (CLARIOstar BMG labtech, analysis software CLARIOstar MARS). Experiments were performed in three biological replicates (n=3).

Western blot

Protein were extracted with RIPA buffer (Santa Cruz, sc 24948A) for 30 minutes on ice with frequent vortex. Samples were sonicated using sonicator (Diagenode BioRuptor) on a 30 s on/off cycle for 3 cycles. Equal amounts of proteins were separated by SDS-PAGE (NUPAGE 4-12% Bis acrylamide NP0335, Invitrogen) and incubated for respective experiments with BMI1 antibody (39993, Actif Motif), RYBP (AB3637, Millipore), CBX8 (sc-374332, Santa Cruz Biotechnology), PRP6 (sc-166889, Santa Cruz Biotechnology), ADAMTS1(12897, CST), MYC(sc-42, Santa Cruz Biotechnology), MMP14(AB53712, Abcam), JNK(44-690G, Thermo Scientific), APOE (sc-390925, Santa Cruz Biotechnology), TNF (Ab9635, Abcam). Tubulin (Sigma, T6199) was used as housekeeping proteins for loading control. Results were visualised using a (BIORAD ChemiDoc MP Imaging). Protein quantification was performed using Fiji image analysis software. Western blots were repeated in three biological replicates per experiment (n=3).

Supplementary table 1

CRISPR plasmid	Addgene reference
**pHR-SFFV-KRAB-dCas9-P2A-mCherry**	60954
**dCAS9-VP64_GFP**	61422
**pKLV-U6gRNA(BbsI)-PGKpuro2ABFP**	50946

Supplementary table 2

Sg	BMI1 nucleotide sequence	Primer Forward	Primer Reverse
**Sg-BMI1-1**	CTCTGCCTT CAGCGGTGC AT	CACCGCTCTGCC TTCAGCGGTGCAT	AAACATGCACCGCTG AAGGCAGAG
**Sg-BMI1-2**	GGAGCACGT GACCCGCTG GG	CACCGGGAGCAC GTGACCCGCTGG G	AAACCCCAGCGGGT CACGTGCTCC
**Sg-BMI1-3**	AGTATGAGA GGCAGAGAT CG	CACCGAGTATGA GAGGCAGAGATC G	AAACCGATCTCTGCC TCTCATACT
**Sg-BMI1-4**	CCGCTCGCA CGCACACAC AC	CACCGCCGCTCG CACGCACACACA C	AAACGTGTGTGTGC GTGCGAGCGG
**Sg-BMI1-5**	GGAGGAGGA GGAGGCCCC GG	CACCGGGAGGAG GAGGAGGCCCCG G	AAACCCGGGGCCTC CTCCTCCTCC

Supplementary table 3

Plasmid	horizon Dharmacon reference
**GIPZ**	RHS4531
**ShBMI1 GIPZ**	RHS4531-EG648
**ShCBX8 GIPZ 367**	RHS4531 V2LHS 247367
**ShCBX8 GIPZ 819**	RHS4531 V2LHS 58819
**ShRYBP GIPZ 667**	RHS4531 V2LHS 24`667
**ShRYBP GIPZ 937**	RHS4531 V3LHS 398937
**ShRYBP GIPZ 177**	RHS4531 V2LHS 20177
**ShPRP6 GIPZ 976**	RHS4430 V2LHS_42976
**ShPRP6 GIPZ 115**	RHS4430 V3LHS_637115
**ShPRP6 GIPZ 119**	RHS4430 V3LHS_637119

References

Gilbert, Luke A., Horlbeck, Max A., Adamson, B., Villalta, Jacqueline E., Chen, Y., Whitehead, Evan H., Guimaraes, C., Panning, B., Ploegh, Hidde L., Bassik, Michael C*., et al.* (2014). Genome-Scale CRISPR-Mediated Control of Gene Repression and Activation. Cell *159*, 647-661.

Koike-Yusa, H., Li, Y., Tan, E.P., Velasco-Herrera Mdel, C., and Yusa, K. (2014). Genome-wide recessive genetic screening in mammalian cells with a lentiviral CRISPR-guide RNA library. Nat Biotechnol *32*, 267-273.

Konermann, S., Brigham, M.D., Trevino, A.E., Joung, J., Abudayyeh, O.O., Barcena, C., Hsu, P.D., Habib, N., Gootenberg, J.S., Nishimasu, H*., et al.* (2015). Genome-scale transcriptional activation by an engineered CRISPR-Cas9 complex. Nature *517*, 583-588.

Ran, F.A., Hsu, P.D., Wright, J., Agarwala, V., Scott, D.A., and Zhang, F. (2013). Genome engineering using the CRISPR-Cas9 system. Nat Protoc *8*, 2281-2308.

Tang, Y., Garson, K., Li, L., and Vanderhyden, B.C. (2015). Optimization of lentiviral vector production using polyethylenimine-mediated transfection. In Oncol Lett, pp. 55-62.

Xie, Y., Bergström, T., Jiang, Y., Johansson, P., Marinescu, V.D., Lindberg, N., Segerman, A., Wicher, G., Niklasson, M., Baskaran, S*., et al.* (2015). The Human Glioblastoma Cell Culture Resource: Validated Cell Models Representing All Molecular Subtypes. EBioMedicine *2*, 1351-1363.

SUPPLEMENTARY FIGURE LEGENDS

Figure S1. Characterisation of BMI1 and RYBP proteome in GBM.

(A) BMI1 transcript levels relative to ACT1 in GBM cell lines and iNSC. (**B**) Heatmap indicating A.U. of integral fluorescence intensity of RNA levels of BMI1 in primary GIC obtained from the HGCC collection. (**C**) Western blot showing expression levels of BMI1 in selected GBM cell lines (U87MG, LN428), primary GIC (U3118 and U3082), foetal NSC and medulloblastoma primary cells ICb1299 as well as adult brain (A). (**D**) BMI1-IP followed by Western blot and MS2 intensities obtained by MS showing specificity of binding in the presence or absence of BMI1 antibody. (**E**) Venn diagram showing proteins shared between the BMI1-interactome in GBM and published RING1B interactome datasets in mESC and mNPC with the network PcG protein complex identified from the 18 proteins shared by all datasets. (**F**) Venn diagram indicating RYBP interactors and associated networks identified within the shared proteins: ncPRC1 complex (blue) and RNA III polymerase cluster (violet). (**G**) Boxplot depicting expression of selected genes in GBM (n= 148) versus non tumour samples (n=4) in the TCGA datasets (Tukey's honest significant differences, *p<0.05, **p<0.005). (**H**) Venn diagram indicating RYBP-only interactors with RNA III polymerase (violet) as the main network. (**I**) RT-PCR showing upregulation on BMI1 expression in in U87MG^{aCRBMI1} vs U87MG^{aCRempty} (n=3, t-test, ** p< 0.05, **p<0.01). (**J**) Growth curves showing impact of BMI1 modulation on proliferation in LN428^{iCRBMI1} vs LN428^{iCRempty}. Fold change indicates a decrease in cell proliferation over seeded cells every 24 hour time point (n=3; 2way ANOVA, *p<0.05, **p<0.01, ***p<0.001) (**K**) RT-PCR showing downregulation on BMI1 expression in LN428^{iCRBMI1} vs LN428^{iCRempty} (n=3, t-test, * p< 0.05, **p<0.01). (**L**) Growth curves showing impact of BMI1 modulation on proliferation in U87MG^{aCRBMI1} vs U87MG^{aCRempty}. Fold change indicates an increase in cell proliferation over seeded cells every 24 hour time point (n=3, 2way ANOVA, *p<0.05). (**M**) Upset plot comparing the BMI1-interactome and its specifically modulated fraction with the RING1B interactome in mESC and mNPC from published datasets.

Figure S2. Characterisation of BMI1 and RYBP chromatome in GBM.

(A) Venn diagram identifying the RYBP chromatome shared between GBM cell lines upon RYBP ChIP-MS (blue). Note CBX8 from the PcG protein complex (blue) is associated to RYBP. (B) Box plot showing expression of selected genes in GBM (n=148) versus non tumour samples (n=4) in the TCGA datasets (Tukey's honest significant differences, *p<0.05, **p<0.005). (C) Venn diagram indicating chromatin-bound proteins upon H2AK119ub ChIP-MS, which are shared between GBM cell lines (blue). (D) Box plot showing expression of selected genes in GBM (n=148) versus non tumour samples (n=4) in TCGA datasets (Tukey's honest significant differences, *p<0.05, **p<0.005). (E) Upset plot comparing BMI1-interactome and BMI1-chromatome with the RING1B interactome in mESC and mNPC from published datasets.

Figure S3. Validation of MS-detected interactions in primary GIC lines.

(**A**) BMI1-IP western blot for BMI1 and CBX8 (representative images) in GBM cell lines and upon CRISPR-editing with quantification (n=3, paired t-test, two-tailed, *p<0.05). (**B**) BMI1-IP western blot for BMI1 and CBX8 (representative images) in primary GIC (n=3, paired t-test, two-tailed, *p<0.05). (**C**) PLA assay to confirm BMI1 and RYBP interaction with CBX8 in GBM cell lines. (**D**) PLA assay to confirm BMI1 and RYBP interaction with CBX8 in GIC. (**E**) Violin plots indicating apoptosis measurements upon BMI1, RYBP or CBX8 silencing in two different GIC (U3118 and U3082) (n=4, 2way ANOVA, ns, not significant).

Figure S4. Characterisation of PRP6 biological function in GBM

(**A**) BMI1-IP western blot for PRP6 and BMI1 (representative images) in GBM cell lines and CRISPR-edited GBM cell lines (n=3). (**B**) BMI1-IP western blot for PRP6 and BMI1 (representative images) in GIC (n=3). (**C**) Western blot (representative image) showing downregulation of PRP6 in 3082^{Sble} vs 3082^{ShPRP6} (**D**) Heatmap showing significantly enriched dysregulated molecular functions (IPA software) upon BMI1 or PRP6 silencing (- log10 of p-value<0.05, P-score = or < 1,3) (**E**) Growth curve graphs showing no impact on cell proliferation upon PRP6 silencing in two different GIC (U3118 and U3082) (n=3 2way ANOVA, ns, not significant). Fold change indicates cell proliferation over seeded cells at 24 hour time point (**F)** Representative images of cell aggregation 7 days afterPRP6 silencing (n=3, 2way ANOVA, ns, not significant). Scale bar is 100 µm. (**G**) Violin plots representing apoptosis measurement upon PRP6 silencing in two different GIC (U3118 and U3082) (n=4 2way ANOVA, ns, not significant). (**H**) Translation elongation (red) and cholesterol biosynthesis (blue) are networks associated to BMI1 AS genes and GBM AS genes from published datasets. (**I**) Structural constituent of ribosome (red), translation initiation (green) and alternative splicing (blue) are networks associated to PRP6 AS genes and GBM AS genes from published datasets.

Figure S5. **BMI1 and mRNA splicing in the regulation of cholesterol synthesis and transport.**

(**A**) Enrichment of cholesterol pathways in DEG upon BMI1 silencing. (**B**) Enzymes involved in the production of cholesterol upon BMI1 silencing. (**C**) Plot showing exon expression and DEU in FDFT1 and HMGCS1 upon BMI1 silencing. (**D**) Cell viability assay (relative luminescence units, RLU) in LN428iCRempty vs LN428iCRBMI1 in the presence of 5 µM DMSO, 2.5 Mm U1866A (U18), 80 ng/ml Simvastatin (Sim) and in the combination of 2.5 mM U1866A (U18) and 80 ng/ml Simvastatin (Sim) respectively (n=3; 2way ANOVA, ns, not significant, *p<0.05, **p<0.01, ***p<0.001). (**E**) Cell viability assay (relative luminescence units, RLU) in LN428aCRempty vs LN428aCRBMI1 in the presence of 5 µM DMSO, 2.5 mM U1866A (U18), 80 ng/ml Simvastatin (Sim) and the combination of 2.5 mM U1866A (U18) and 80 ng/ml Simvastatin (Sim) respectively (n=3; 2way ANOVA, ns, not significant, *p<0.05, **p<0.01, ***p<0.001).

Freire-Beneitez et al. Figure S1

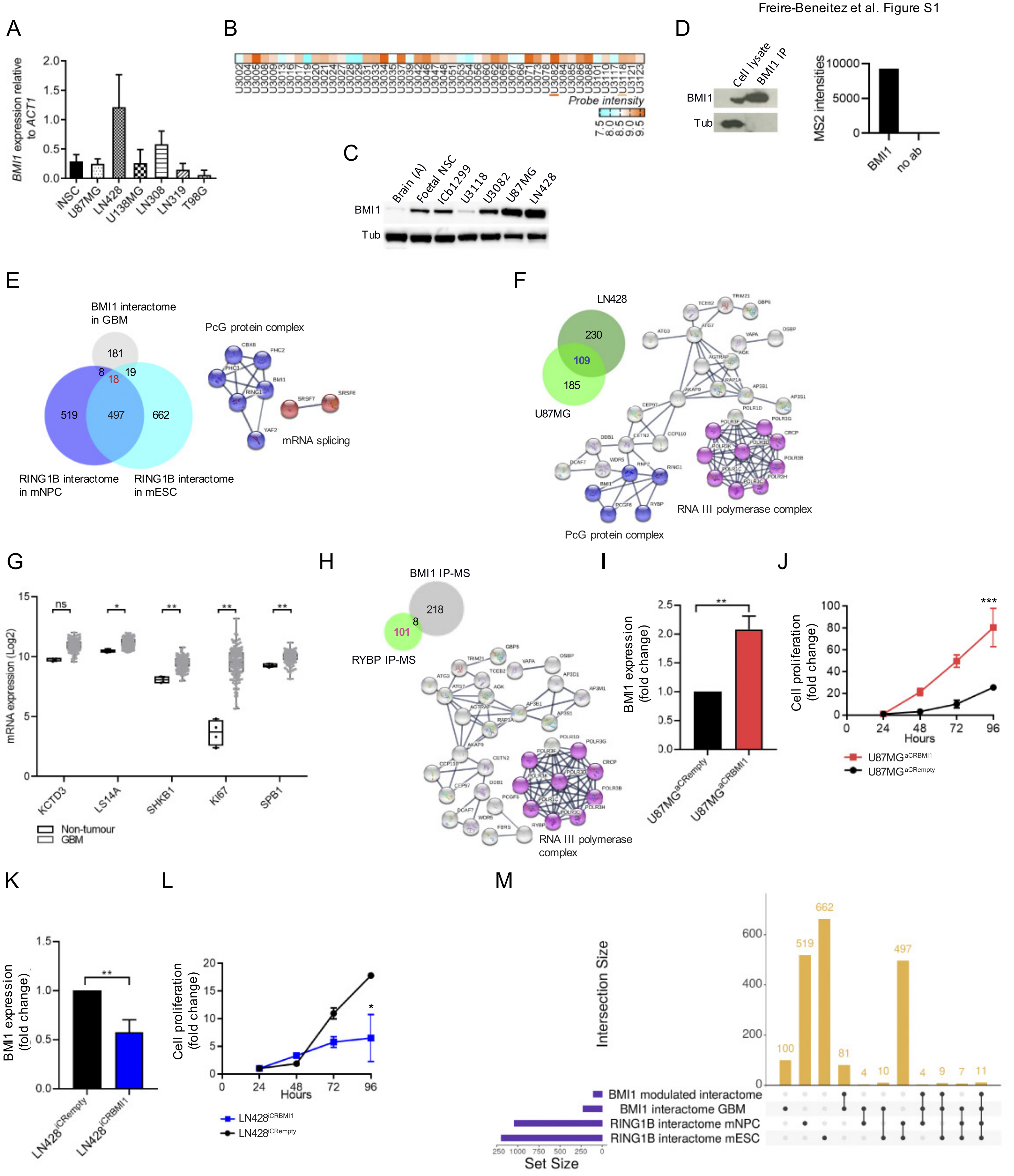

Freire-Beneitez et al. Figure S2

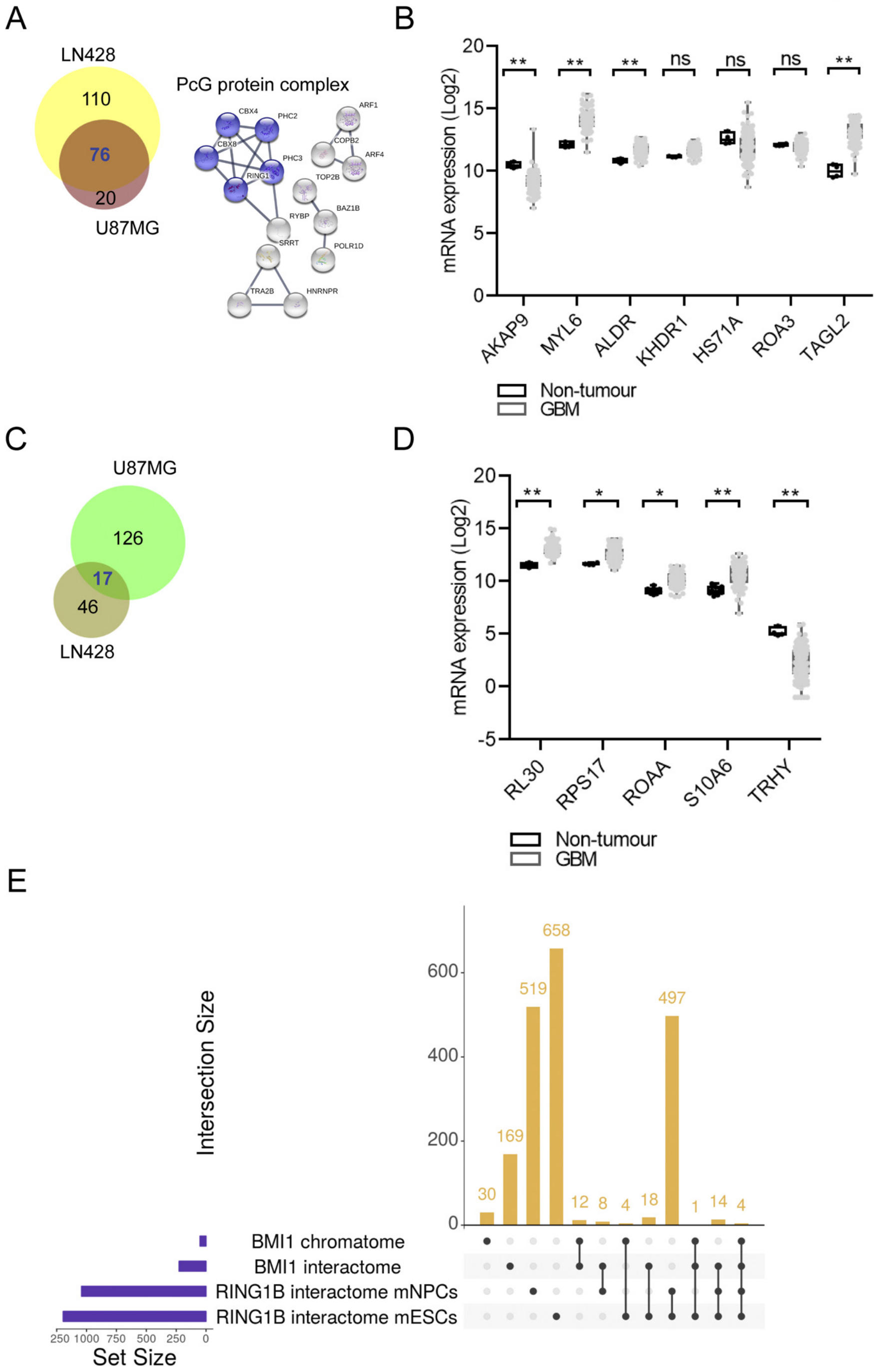

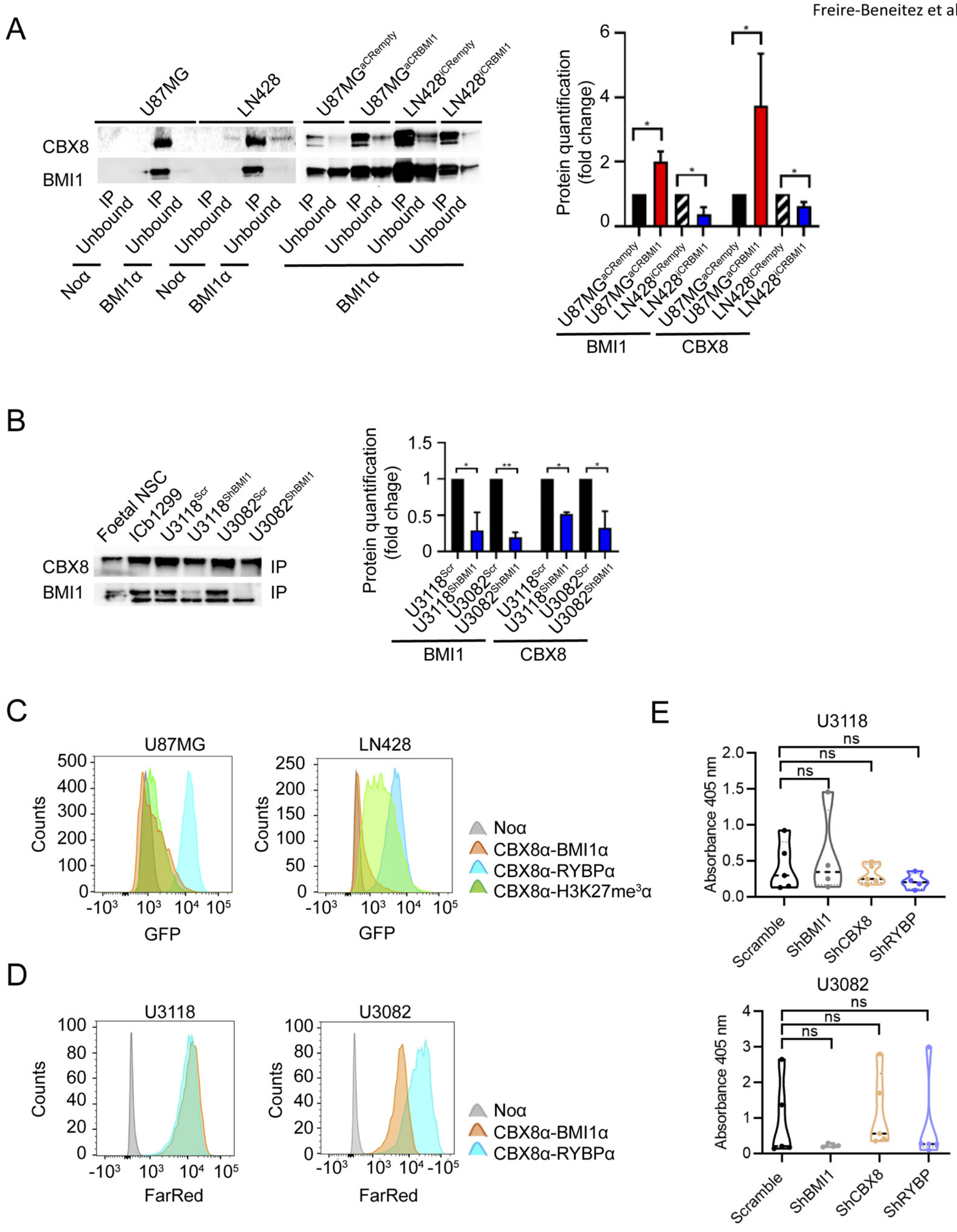
A
U87MG
LN428
CBX8
BMI1
IP
Unbound
Noα
BMI1α
Protein quantification (fold change)
BMI1
CBX8
B
Foetal NSC
ICb1299
CBX8
BMI1
IP
Protein quantification (fold chage)
BMI1
CBX8
C
U87MG
LN428
Counts
GFP
Noα
CBX8α-BMI1α
CBX8α-RYBPα
CBX8α-H3K27me3α
D
U3118
U3082
Counts
FarRed
Noα
CBX8α-BMI1α
CBX8α-RYBPα
E
U3118
Absorbance 405 nm
ns
Scramble
ShBMI1
ShCBX8
ShRYBP
U3082

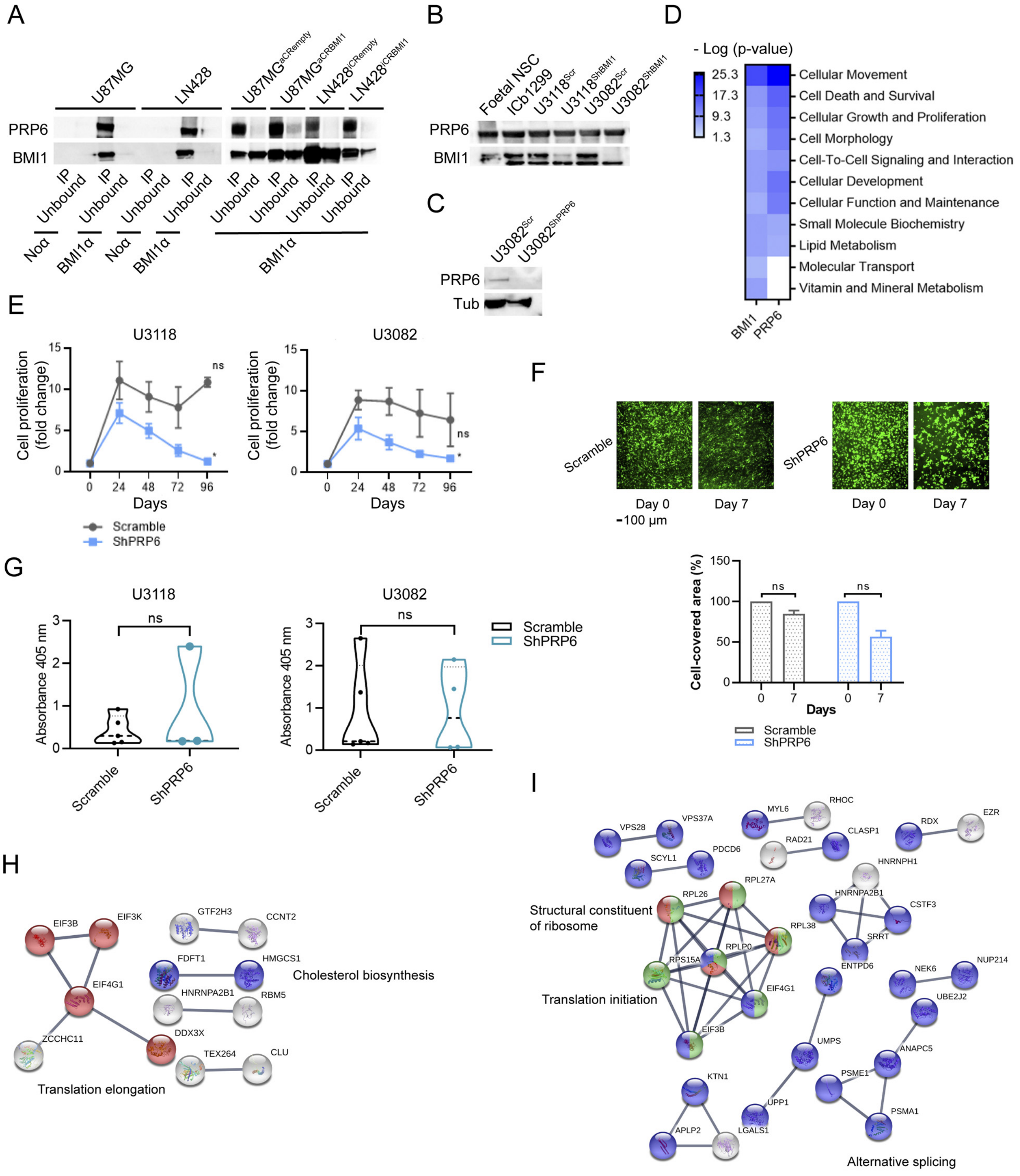
A
U87MG
LN428
PRP6
BMI1
IP
Unbound
Noα
BMI1α
B
Foetal NSC
ICb1299
PRP6
BMI1
C
PRP6
Tub
D
- Log (p-value)
25.3
17.3
9.3
1.3
Cellular Movement
Cell Death and Survival
Cellular Growth and Proliferation
Cell Morphology
Cell-To-Cell Signaling and Interaction
Cellular Development
Cellular Function and Maintenance
Small Molecule Biochemistry
Lipid Metabolism
Molecular Transport
Vitamin and Mineral Metabolism
BMI1
PRP6
E
U3118
U3082
Cell proliferation (fold change)
Days
Scramble
ShPRP6
ns
F
Scramble
ShPRP6
Day 0
Day 7
100 μm
Cell-covered area (%)
Days
G
U3118
U3082
Absorbance 405 nm
Scramble
ShPRP6
ns
H
EIF3B
EIF3K
EIF4G1
ZCCHC11
DDX3X
GTF2H3
CCNT2
FDFT1
HMGCS1
HNRNPA2B1
RBM5
TEX264
CLU
Cholesterol biosynthesis
Translation elongation
I
VPS28
VPS37A
MYL6
RHOC
RDX
EZR
RAD21
CLASP1
SCYL1
PDCD6
HNRNPH1
RPL27A
RPL26
HNRNPA2B1
CSTF3
RPL38
RPLP0
SRRT
RPS15A
ENTPD6
NEK6
NUP214
EIF4G1
UBE2J2
EIF3B
UMPS
ANAPC5
PSME1
KTN1
UPP1
PSMA1
APLP2
LGALS1
Structural constituent of ribosome
Translation initiation
Alternative splicing

Freire-Beneitez et al. Figure S5

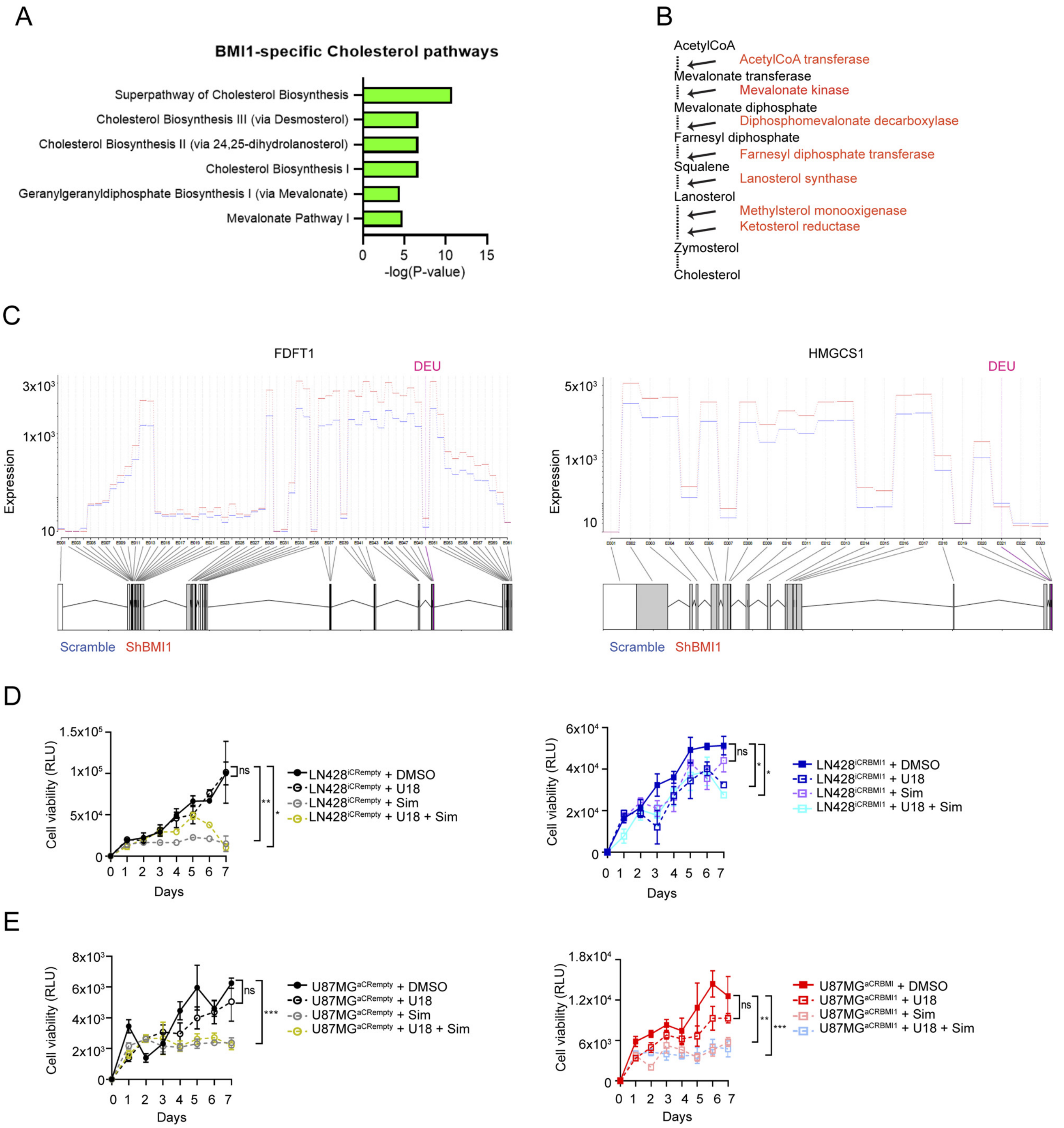

Supplement: zcab009_Supplemental_Files [file zcab009_supplemental_files.zip › Freire et al Suppl Material and Figures Merged File.pdf]
