# Supplementary material for: Elucidation of the BMI1 interactome identifies novel regulatory roles in glioblastoma
Source: NAR Cancer. 2021 Mar 22;3(1):zcab009. doi: 10.1093/narcan/zcab009 (PMC8210184; doi:10.1093/narcan/zcab009)
Supplement: zcab009_Supplemental_Files [file zcab009_supplemental_files.zip › Supplementary Data 10. Certificate of authentication for U87MG cell line.pdf]

Queen Mary University
Blizard Institute, Genomics and Child Health

Veronica Freire Beneitez
The Blizard Building, 4 Newark Street
London, E1 2AT
UK

06.10.2017

# Certificate

**Order**
By order of Veronica Freire Beneitez (Queen Mary University) we were requested to perform a cell line authentication test. Following samples were examined:

| Our sample number | Client sample name |
|---|---|
| CL171005_001 | U-87MG |
| CL171005_002 | LN-428 |

**Method:**
DNA was isolated separately from the samples.
Genetic characteristics were determined by PCR-single-locus-technology. 21 independent PCR-systems Amelogenin, D3S1358, D1S1656, D6S1043, D13S317, Penta E, D16S539, D18S51, D2S1338, CSF1PO, Penta D, TH01, vWA, D21S11, D7S820, D5S818, TPOX, D8S1179, D12S391, D19S433 and FGA were investigated (Promega, PowerPlex 21 PCR Kit).
In parallel, positive and negative controls were carried out yielding correct results.

eurofins | Forensik

DAkkS Deutsche Akkreditierungsstelle D-PL-18779-01-00

CERTIFIED ISO 9001:2008 ACCORDING TO

**Results:**

| DNA-System | DNA-criteria<br>U-87MG<br>CL171005_001 | DNA-criteria<br>LN-428<br>CL171005_002 |
|---|---|---|
| AM | X, X | X, Y |
| D3S1358 | 16, 17 | 16, 17 |
| D1S1656 | 15, 15 | 16.3, 16.3 |
| D6S1043 | 11, 18 | 18, 20 |
| D13S317 | 11, 11 | 8, 8 |
| Penta E | 7, 14 | 14, 16 |
| D16S539 | 12, 12 | 9, 9 |
| D18S51 | 13, 13 | 13, 17 |
| D2S1338 | 20, 23 | 17, 17 |
| CSF1PO | 10, 11 | 10, 10 |
| Penta D | 9, 14 | 13, 13 |
| TH01 | 9.3, 9.3 | 8, 9.3 |
| vWA | 15, 17 | 16, 16 |
| D21S11 | 28, 32.2 | 30, 31 |
| D7S820 | 8, 9 | 8, 12 |
| D5S818 | 11, 12 | 11, 13 |
| TPOX | 8, 8 | 8, 11 |
| D8S1179 | 10, 11 | 12, 13 |
| D12S391 | 18, 21 | 17, 18 |
| D19S433 | 15, 15.2 | 14, 15 |
| FGA | 18, 24 | 20, 25 |

**Summary:**

The following cell lines could be detected in the online database of the DSMZ (http://www.dsmz.de/de/service/services-human-and-animal-cell-lines/online-str-analysis.html):

| Our sample number | Client sample name | DSMZ name |
|---|---|---|
| CL171005_001 | U-87MG | U-87MG |
| CL171005_002 | LN-428 | LN-428 (expasy.org) |

Dr. Burkhard Rolf
Director Forensic Services

Dr. Michaela Bosch
Project Manager DNA-Forensics

Eurofins Medigenomix Forensik GmbH carries out all analyses with greatest care and on the basis of state of the art scientific knowledge. All results solely refer to the analysed samples. Our expert's reports must not be duplicated in extracts without consent of Eurofins Medigenomix Forensik GmbH.
Cell_line-certificate_eng_V05_21032017

Vorlage_Verwandtschaftsanalyse_EUROFINS_v02_121127

Eurofins Medigenomix Forensik GmbH
Anzinger Str. 7a
D-85560 Ebersberg

Tel. +49 (0) 8092 8289 400
Fax +49 (0) 8092 8289 201
www.eurofins.de/forensik-de
forensik@eurofins.com

Geschäftsführer
Dr. Peter Persigehl
Dr. Bruno Poddevin

HRB 20 12 60
Amtsgericht München

Ust-Id Nr. DE 285161076
Steuernummer 114/116/00159
