# Supplementary material for: Elucidation of the BMI1 interactome identifies novel regulatory roles in glioblastoma
Source: NAR Cancer. 2021 Mar 22;3(1):zcab009. doi: 10.1093/narcan/zcab009 (PMC8210184; doi:10.1093/narcan/zcab009)

Elektropherogramm

F06_CL171005_001_CE_17_0427_005.fsa (CL171005_001, 2017/27504)

Promega PowerPlex 21 (WEN ILS 500)

Ct Gesamt: 22,87 Qty Gesamt: 3,16

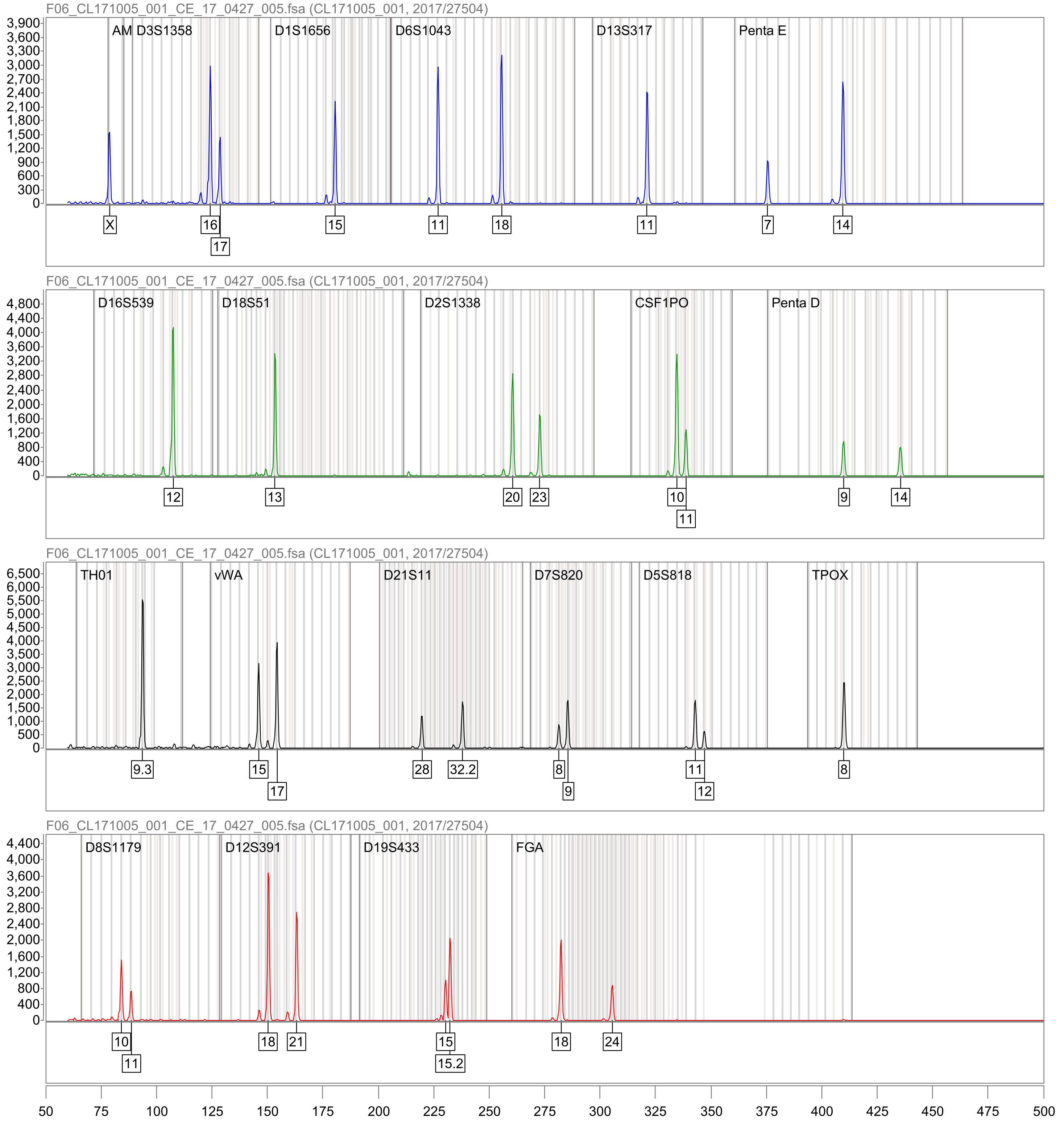

Elektropherogramm

H06_CL171005_002_CE_17_0427_007.fsa (CL171005_002, 2017/27504)

Promega PowerPlex 21 (WEN ILS 500)

Ct Gesamt: 23,1 Qty Gesamt: 2,63

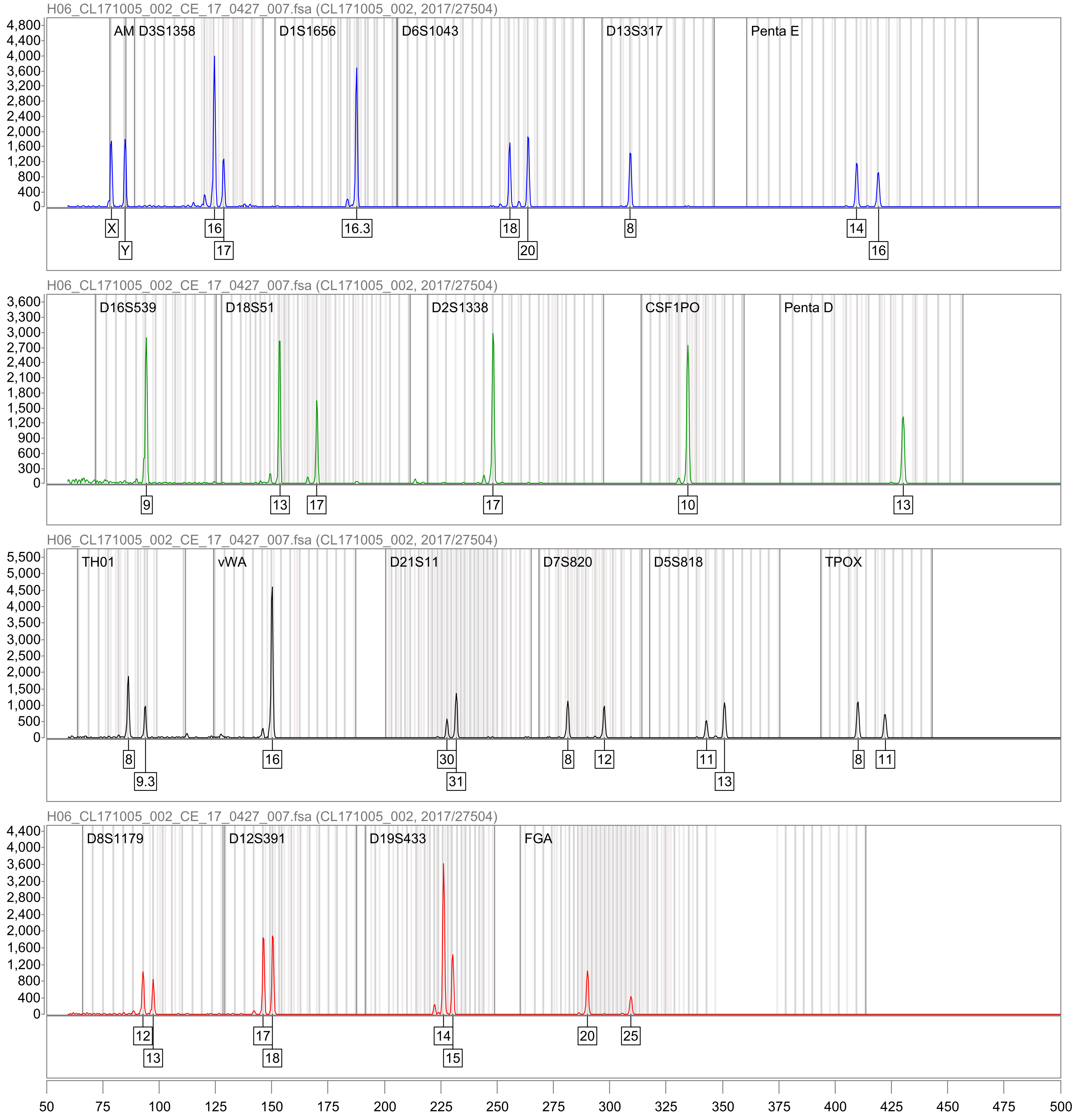

Supplement: zcab009_Supplemental_Files [file zcab009_supplemental_files.zip › Supplementary Data 11. Elektropherogramm of authentication for U87MG and LN428 cell lines..pdf]
